# Inducible CRISPR/Cas9 Allows for Multiplexed and Rapidly Segregated Single-Target Genome Editing in *Synechocystis* Sp. PCC 6803

**DOI:** 10.1021/acssynbio.2c00375

**Published:** 2022-08-15

**Authors:** Ivana Cengic, Inés C. Cañadas, Nigel P. Minton, Elton P. Hudson

**Affiliations:** † School of Engineering Sciences in Chemistry, Biotechnology and Health, Science for Life Laboratory, KTH Royal Institute of Technology, Stockholm 17121, Sweden; ‡ BBSRC/EPSRC Synthetic Biology Research Centre (SBRC), School of Life Sciences, The University of Nottingham, Nottingham NG7 2RD, U.K.

**Keywords:** CRISPR, Cas9, cyanobacteria, inducible, riboswitch, multiplex

## Abstract

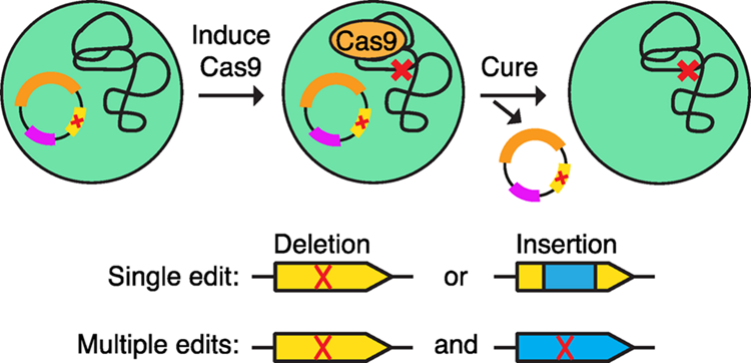

Establishing various synthetic biology tools is crucial for the development of cyanobacteria for biotechnology use, especially tools that allow for precise and markerless genome editing in a time-efficient manner. Here, we describe a riboswitch-inducible CRISPR/Cas9 system, contained on a single replicative vector, for the model cyanobacterium *Synechocystis* sp. PCC 6803. A theophylline-responsive riboswitch allowed tight control of Cas9 expression, which enabled reliable transformation of the CRISPR/Cas9 vector into*Synechocystis*. Induction of the CRISPR/Cas9 mediated various types of genomic edits, specifically deletions and insertions of varying size. The editing efficiency varied depending on the target and intended edit; smaller edits performed better, reaching, e.g., 100% for insertion of a FLAG-tag onto *rbcL*. Importantly, the single-vector CRISPR/Cas9 system mediated multiplexed editing of up to three targets in parallel in*Synechocystis*. All single-target and several double-target mutants were also fully segregated after the first round of induction. Lastly, a vector curing system based on the nickel-inducible expression of the toxic *mazF* (from *Escherichia coli*) was added to the CRISPR/Cas9 vector. This inducible system allowed for curing of the vector in 25–75% of screened colonies, enabling edited mutants to become markerless.

## Introduction

Cyanobacteria are photosynthetic prokaryotes that have gained interest as cell factories for sustainable production of various compounds.^1−3^ However, in order for large-scale processes to become economically feasible, further study and significant engineering of these cyanobacterial hosts is required. While the toolset to engineer cyanobacteria is steadily growing,^4−6^ there is still need for reliable tools that allow for precise, markerless, rapid, and multiplexed editing in these polyploid organisms^7^ that are commonly time-consuming to engineer.

The CRISPR/Cas genome editing system is one such promising tool.^8−12^ A guide RNA (gRNA) is provided to guide the Cas endonuclease to a specific complementary target DNA site (the protospacer). This protospacer target must be next to a protospacer adjacent motif (PAM). Binding of the Cas-gRNA effector complex to the target DNA results in a double-stranded break (DSB) that is lethal for the cell unless mended. The most common method to mend DSBs in prokaryotes is by homology-directed repair (HDR), whereby a donor DNA is provided as a repair template. The Cas protein first popularized for widespread use was the class 2, type II Cas9 protein from *Streptococcus pyogenes*.^9,13^ However, besides being an efficient endonuclease it can also be cytotoxic when overexpressed, both alone and particularly when coexpressed with a single guide RNA (sgRNA), resulting in low transformation efficiencies and few edited transformants.^14^ Some strategies to circumvent Cas9 toxicity are to identify and use alternative endonucleases, to engineer endogenous bacterial CRISPR systems, or to decouple the transformation and editing steps by having inducible expression of Cas9.^14^

Several CRISPR/Cas systems have been described for cyanobacteria. In the two first studies, performed in *Synechococcus* UTEX 2973 and *Synechococcus elongatus* PCC 7942, transient expression of Cas9 supported editing, although cytotoxicity was a noted issue.^15,16^ In a later study, constitutive expression of the less toxic Cpf1 (also named Cas12a) endonuclease proved useful to mediate various types of edits (point mutation, knock-out, and knock-in) in UTEX 2973, *Synechocystis* sp. PCC 6803 (hereafter S6803), and *Anabaena sp.* PCC 7120.^17^ However, several passages on selective media were required to increase the percentage of edited and fully segregated colonies, especially in the highly polyploid S6803. This Cpf1-system was further evaluated in *Anabaena*, where it facilitated sequential and simultaneous deletion of two genes, and was supplemented with a counter-selection tool to cure edited cells of the Cpf1-plasmid.^18^ Only one example of an inducible CRISPR/Cas9 system has, to our knowledge, been described for cyanobacteria. There, *cas9* was integrated into the genome of the S6803 host and its expression was controlled by an aTc-inducible promoter.^19^ However, leaky Cas9 expression in uninduced cells caused low transformation efficiencies (∼10 CFU/μg DNA) for sgRNA-expressing plasmids. An alternative method using site-specific recombinases has also been used to make markerless genome edits in *Synechococcus* sp. PCC 7002 and S6803;^20^ however, this method still leaves short recombination scars at the edit sites unlike CRISPR/Cas9 which supports scarless edits.

Development of tightly controlled CRISPR/Cas9 systems has until now been more successful in other types of bacteria.^14,21−23^ While many studies have used the traditional pairing of inducible promoter and transcriptional regulator, several recent studies have successfully employed riboswitches as regulatory elements for Cas9 expression.^24−26^ Riboswitches are small structured RNA elements commonly found in the 5’-UTR of mRNAs.^27,28^ Their small size is especially useful when building CRISPR/Cas systems contained on single plasmids. The aptamer domain of a riboswitch is able to selectively bind to a specific ligand, triggering a conformational change that affects the expression platform domain and thus regulates the expression of the downstream gene.^27^ Often this regulation occurs at the transcriptional or translational level.

The riboswitches used to control CRISPR/Cas9 systems in bacteria have been based mainly on the synthetic theophylline-specific aptamer.^29^ This aptamer was used by Topp et al. to develop a set of six theophylline-inducible riboswitches (A – E + E*), widely applicable in different bacteria.^30^ These riboswitches regulate the expression of a downstream gene at the translational level by blocking access to the mRNA’s ribosome binding site (RBS) when no theophylline ligand is bound. When theophylline binds, the RBS is made available and translation can proceed. These theophylline riboswitches have already been evaluated in various cyanobacteria,^31−34^ and used in developing tools such as NOT gates in, e.g., S6803,^33^ inducible protein degradation systems in *S. elongatus* PCC 7942,^35,36^ and inducible CRISPR-interference systems in S6803 and *Anabaena*.^37,38^ In this study, they were further used to design a tightly controlled CRISPR/Cas9 system for inducible genome editing in S6803.

## Results and Discussion

### Designing the Riboswitch-Based Inducible CRISPR/Cas9 System for S6803

The goal of this study was to build a tightly controlled CRISPR/Cas9 system for S6803, where the expression of Cas9, and therefore genome editing, is controlled by a theophylline-inducible riboswitch. This system was built to be self-contained on a single plasmid based on the replicative pPMQAK1-T vector with an RSF1010-replicon.^39,40^

Of note is that the S6803 host used in this study is highly polyploid, with, on average, 20 genome copies observed during exponential growth.^7^ Any promising CRISPR/Cas9 system must therefore be able to edit all genomic copies of the intended target DNA to produce a fully segregated mutant. As the level of Cas9 required to achieve such editing in S6803 is unknown, three different riboswitches from the set developed by Topp et al.^30^ were tested to control its expression. The aim was to identify which one would provide the correct balance of nonleaky Cas9 expression when un-induced, allowing high transformation efficiency of the CRISPR/Cas9 vector, and high enough expression when induced to allow for successful genome editing. The two riboswitches shown to have the least leaky expression in S6803, B and C,^33^ were selected. The more leaky variant E* was also selected,^33^ to include a riboswitch that supports a higher induced expression level if needed.

In a preliminary part of this study, these riboswitches were combined with the *conII* promoter (P_*conII*_) as has been done in several cyanobacteria studies.^32,33^ However, no useful system resulted from these efforts, which is mainly due to inefficiently induced CRISPR/Cas9 genome editing (data not shown). In a new attempt, the same riboswitches were instead combined with the *trc* promoter (P_*trc*_) as described by Nakahira et al.;^31^ this also meant adding the P_*trc*_ 5′-UTR and a constant region upstream of the riboswitches. Note that the LacI repressor was not expressed in any of the strains in this study, so the inducibility of P_*trc*_ was only due to its combination with the riboswitches. To ensure that the theophylline concentrations used to induce the riboswitches would not be toxic, its toxicity toward S6803 was evaluated (Figure S1). At 0.5 mM theophylline, there was no apparent growth defect, either in culture or on plates.

The effect of changing the promoter and 5’-UTR region upstream of the riboswitches was studied with a Gfp reporter (Figure S2). The P_*trc*_ variants supported higher expression levels and induction ratios than the P_*conII*_ ones. P_*trc*_-[B] and P*_trc_-*[E*] showed maximum induction ratios of 68- and 30-fold at 0.5 mM theophylline, respectively. The higher induction ratio for P_*trc*_-[B] compared to P_*trc*_-[E*] was due to less leaky expression, as P_*trc*_-[E*] allowed a 7-fold higher absolute expression level. In comparison, P_*trc*_-[C] underperformed due to leaky and weak expression and only reached a maximum 14-fold induction at 0.5 mM.

Two strategies to additionally lower the Cas9-levels in uninduced cells were explored. One was to add an *ssrA* protease degradation tag to the C-terminus of Cas9.^21^ The selected tag (LVA) has been estimated to reduce the steady-state level of a tagged protein by 95% in S6803.^39,41^ The other strategy was to mutate the −10-box in P_*trc*_ to emulate −10 boxes found in weaker promoters.^42^ The P_*trc*_ shares the same −10-box (TATAAT) as the strong BioBrick BBa_J23119 promoter, which belongs to a promoter library that has been evaluated in S6803.^40^ Promoters from this library that drive weaker expression and that only differ in their −10-box sequence were used to select the alternative −10-boxes. These selected ones came from promoters BBa_J23104 (TATTGT), BBa_J23116 (GACTAT), and BBa_J23117 (GATTGT). These were hereafter identified by their last three digits (104, 116, 117) and were estimated to weaken P_*trc*_ by roughly 65%, 90%, and 99%, respectively. These two weakening strategies were applied separately from each other to all P_*trc*_ riboswitch (B, C, or E*) pairs.

**Figure 1:**
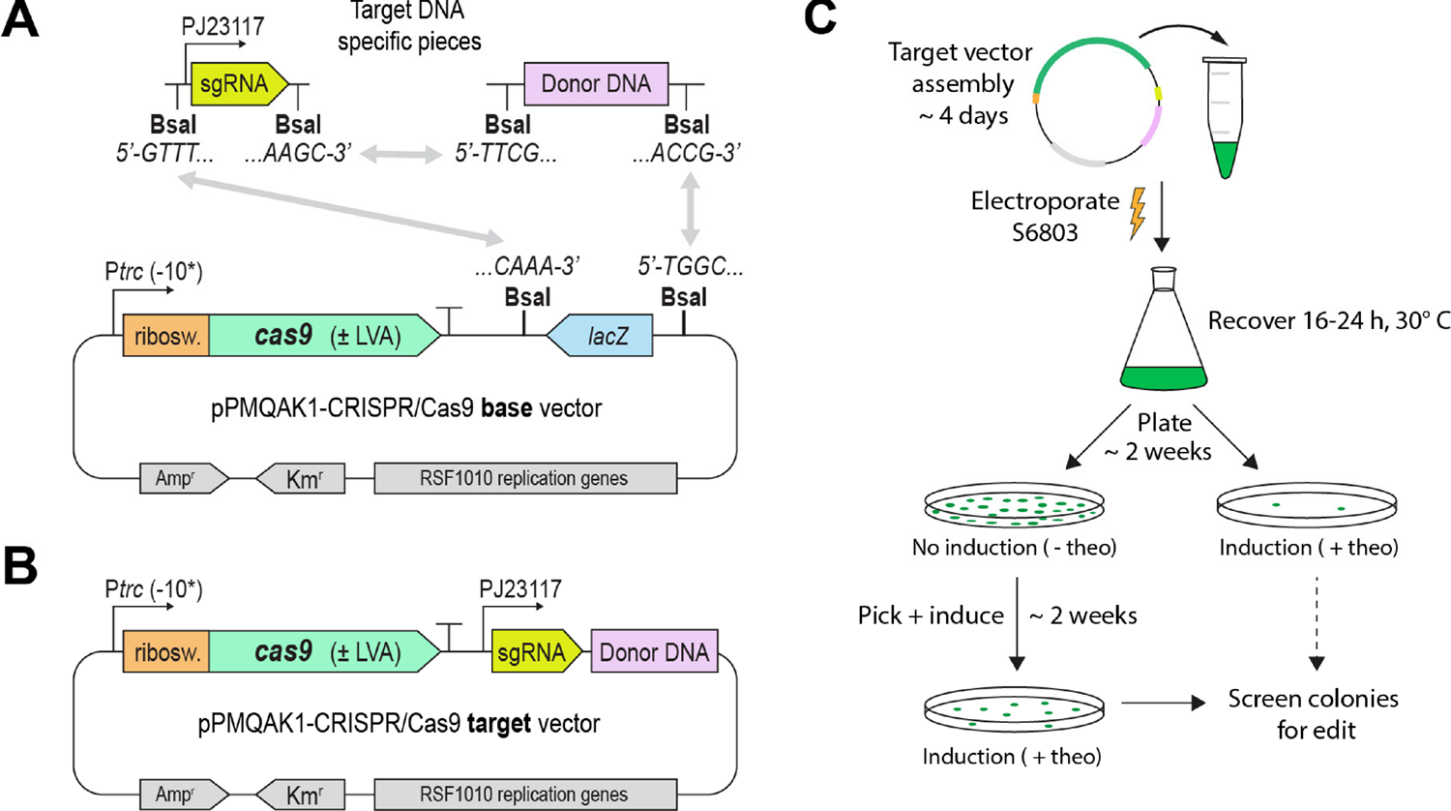
Overview of the pPMQAK1-CRISPR/Cas9 vector build and general workflow. (A) Schematic showing the pPMQAK1-CRISPR/Cas9 base vector and target DNA-specific pieces, i.e., sgRNA and donor DNA, with the BsaI overhangs required for one-step Golden Gate assembly. The vector variants differ in their expression of *cas9*: one of three riboswitches (“ribosw.”) mediate induction with theophylline, P_*trc*_ has −10-boxes with differing strengths (−10*), and some variants include an LVA-tagged Cas9 (±LVA). (B) Map of the final pPMQAK1-CRISPR/Cas9 target vector.(C) Workflow to transform (by electroporation) a constructed pPMQAK1-CRISPR/Cas9 target vector into S6803 and induce CRISPR/Cas9-mediated editing. Approximate time for each step is indicated.

In total, this created 15 different pPMQAK1-CRISPR/Cas9 base vectors to evaluate. All vectors were constructed in the same way for ease of use; the *cas9* expression cassette was followed by a *lacZ* sandwiched between two Golden Gate cloning BsaI sites (Figures 1a and S3a for a more detailed vector map). The pieces specific to the target DNA, the sgRNA and donor DNA, were constructed with compatible BsaI sites (Figure 1a), enabling simultaneous ligation of both into the base vector to create the final target vector (Figure 1b).

The promoter selected for sgRNA expression was the constitutive but weak BioBrick BBa_J23117.^40^ Weak sgRNA expression was deemed to complement the inducible CRISPR/Cas9 system better, since unnecessary overexpression could exacerbate the action of any leaky Cas9. To select the spacers used in the sgRNAs, the Cas9-specific NGG-PAM was used to search the target DNA area for suitable protospacers. The sgRNAs were designed to target as close to the desired edit site as possible, considering that Cas9 cuts 3–4 nucleotides upstream of the PAM.^12^ A short distance between the DSB and edit site is correlated with better editing efficiency.^43^ The sgRNAs were also constructed to target the template strand (unless noted otherwise); this allows for faster dislodging of the Cas9-sgRNA complex from the cut site, enabling improved access for the HDR machinery.^44^

Depending on the desired genome edit, the donor DNA was altered accordingly. Generally, the donor DNA included 350 bp homology arms on either side of the edit site, and was designed to remove or silently mutate the PAM and proximal protospacer sequence to avoid recognition and continued cutting of the target site after editing.

The workflow (Figure 1c) used in this study took advantage of the decoupling of transformation and genome editing made possible by having inducible Cas9 expression. Plating of transformed, by electroporation, and recovered S6803 on noninducer plates resulted in many transformants for the pPMQAK1-CRISPR/Cas9 target vector. These transformants were subsequently plated on inducer plates to undergo CRISPR/Cas9-mediated genome editing. Surviving colonies were screened for the desired edit. To control that the constructed pPMQAK1-CRISPR/Cas9 target vector was functional in mediating DSBs, i.e., had an efficient sgRNA, half of the transformed and recovered S6803 were plated directly on inducer plates. If the DNA targeting was functional, these yielded no or very few surviving transformants due to the lethality of DSBs and low efficiency of HDR. If any transformants survived, they were screened for the desired edit.

### Proof-of-Principle Test To Identify Promising CRISPR/Cas9 Base Vectors

**Figure 2:**
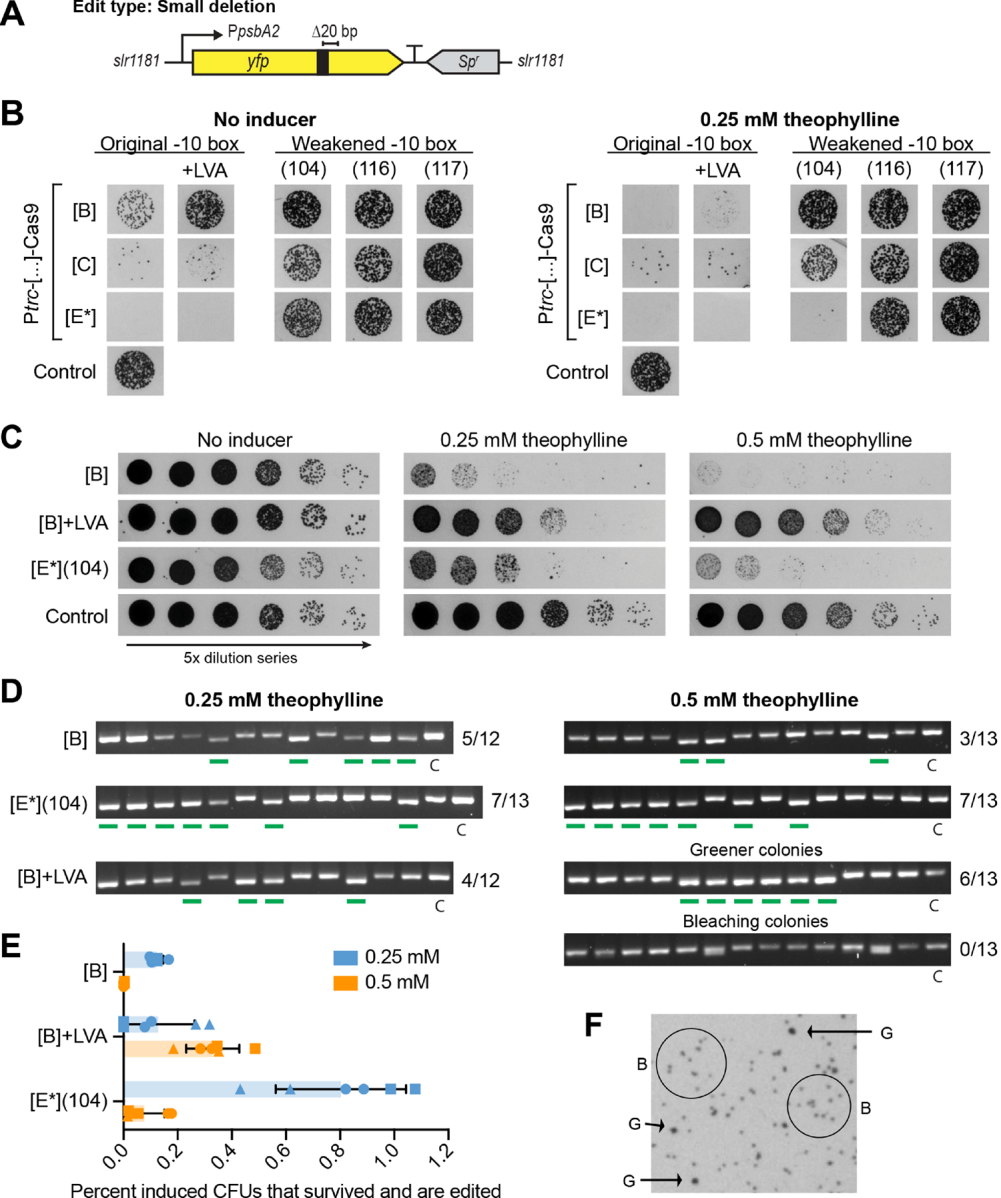
Evaluating the built pPMQAK1-CRISPR/Cas9 vectors in S6803 by performing a small deletion. (A) Schematic of the genomic *yfp* target in S6803 Δ*slr1181*::P_*psbA2*_-Yfp-B0015-Sp^r^, showing approximate placement of the sgRNA target region (black box) and desired 20 bp deletion. (B) Transformation results after plating on selective plates without or with 0.25 mM theophylline inducer. The 15 *yfp*-targeting pPMQAK1-CRISPR/Cas9 vector variants differ in their *cas9*-expression strength, and the P_*trc*_ is fused to riboswitch B, C, or E* and evaluated as is, in combination with a degradation tag (+LVA) on Cas9, or with a weakened P_*trc*_ by changing its original −10-box (strength: original > 104 > 116 > 117). The control lacks *cas9* expression. (C) Induction spot assay results for [B], [B] + LVA, and [E*](104), and the control. 5× dilution series were plated on regular BG11-plates or plates supplemented with 0.25 or 0.5 mM theophylline inducer. Done for biological triplicates in technical duplicates, representative data is shown. (D) Editing results for [B], [B] + LVA, and [E*](104). A green line below a lane signals a fully edited (Δ20 bp) mutant. A control (“C”) shows how an unedited colony will appear. Fractions indicate the number of fully edited colonies out of the total number screened. Two representative colony phenotypes were screened for [B] + LVA induced on 0.5 mM theophylline. (E) Percentage of CFUs that retained a healthy phenotype (i.e., survived) and became edited, after being induced on 0.25 or 0.5 mM theophylline. Bars show the averages ± SD for induced biological triplicates (different symbols, i.e., a circle, triangle, or square, distinguishes the data for each triplicate) done in technical duplicates. (F) Example of the two representative phenotypes found on inducer plates, i.e., healthier, greener colonies (indicated by arrows and marked “G”), and bleaching colonies (circled and marked “B”).

To narrow down the set of the 15 designed pPMQAK1-CRISPR/Cas9 base vectors, a proof-of-principle test was done. The goal was to introduce a small 20 bp deletion into the *yfp* gene of a S6803 Δ*slr1181*::P_*psbA2*_-Yfp-B0015-Sp^r^ strain (Figure 2a). An sgRNA targeting close to the middle of *yfp* was selected, while the desired 20 bp deletion included removal of the GGG-PAM and preceding three bases of the protospacer. The donor DNA contained homology arms surrounding the deletion site. All 15 resulting CRISPR/Cas9 *yfp* target vectors were transformed into strain Δ*slr1181*::P_*psbA2*_-Yfp-B0015-Sp^r^ and treated according to the workflow in Figure 1c. A control vector where *cas9* lacked a promoter and start codon, i.e., resulting in no expression, was also included. This vector served as a control for the toxicity exhibited by any potentially leaky Cas9 expression in the tested vector variants. Its similarly large size (only 160 bp smaller) also worked as a control for the transformation efficiency for such large vectors (∼13 kbp) into S6803.

The desired result was a CRISPR/Cas9 target vector that resulted in many transformants under noninduced conditions but was proven effective in inducing lethal DSBs when induced with theophylline. The transformation results (Figure 2b) clearly singled out constructs P_*trc*_-[B]-Cas9 (hereafter named [B]), P_*trc*_-[B]-Cas9 + LVA (hereafter named [B] + LVA), and P_*trc*_(−10-box:104)-[E*]-Cas9 (hereafter named [E*](104)) as having these desirable traits. Based on the previous Gfp reporter results (Figure S2), and what is known about the modifications introduced to weaken the expression or stability of Cas9 in these constructs, these systems were estimated to rank as follows in terms of resulting Cas9 amounts: [E*](104) > [B] > [B] + LVA. The control showed that 0.25 mM theophylline alone did not affect the transformation efficiency.

Colonies from the transformation plates (Figure S4a) were screened to check for leaky editing in the absence of any inducer, and if the few surviving transformants on the theophylline plates (Figure S4a,b) were indeed edited or had remained unedited by somehow “escaping” the CRISPR/Cas9. While no leaky editing was observed for the uninduced transformants, a fraction of the surviving induced transformants were fully edited, with editing efficiencies of 25–87.5% depending on the construct (Figure S4c). This showcases the tight control of these CRISPR/Cas9 systems and also the possibility of obtaining fully edited transformants directly after electroporation.

To further evaluate the CRISPR/Cas9-induction and *yfp* (Δ20 bp) editing efficiency of the promising [B], [B] + LVA, and [E*](104) constructs, triplicate transformants were induced on plates with theophylline. The promoterless-*cas9* vector was included as a control. In an attempt to titrate the Cas9 expression, induction on 0.25 and 0.5 mM theophylline was compared. The resulting representative spot assays (Figure 2c) expectedly showed widespread cell death due to induction of the CRISPR/Cas9 system. To determine the *yfp* (Δ20 bp) editing efficiency, surviving colonies that appeared healthy on the inducer plates were picked and screened by colony-PCR (Figure 2d). While no construct reached 100% editing efficiency, all edited colonies were fully segregated after just one round of induction. In addition, the editing efficiencies for the two tested theophylline concentrations were comparable, showing that higher induction was not necessary. Overall, the stronger Cas9 expressing construct [E*](104) supported the best *yfp* (Δ20 bp) editing efficiency, reaching 54% for both tested inducer concentrations. To better judge the difference between the two tested theophylline concentrations and CRISPR/Cas9 constructs, the respective editing efficiency for the screened colonies was related to the percentage of CFUs that retained a healthy and green phenotype (i.e., “survived”) after induction (see the Methods section for details). This quantified the total percentage of induced CFUs that survived induction and became edited. The weakest [B] + LVA construct was found to benefit from increased Cas9 expression at 0.5 mM theophylline (Figure 2e). Meanwhile, induction with 0.25 mM outperformed 0.5 mM for constructs [B] and [E*](104) (Figure 2e), due to fewer cells surviving on the higher concentration (Figure 2c). Taken together, the decision was made to use the lower 0.25 mM theophylline concentration for the rest of this study.

Among the colonies that appeared on plates after CRISPR/Cas9-induction, two different phenotypes were observed. One type was greener and larger, while the second was smaller and slightly bleached (Figure 2f). This second phenotype was more prevalent for the weakest [B] + LVA construct and was responsible for the apparent higher survival of these cells on theophylline (Figure 2c). When these two colony types were compared in terms of *yfp* (Δ20 bp) editing for construct [B] + LVA, only the larger, greener ones exhibited any fully segregated edits (Figure 2d). Also, after prolonged induction on theophylline the smaller colonies were found to bleach entirely and die (Figure S4d), likely due to incomplete or absent editing and continued exposure to lethal Cas9-catalyzed DSBs. This phenotypic difference, if prevalent after induction, can therefore be useful when choosing which obtained colonies are to be screened. This strategy was applied throughout this continued study; only the greener and therefore healthier-looking colonies were assessed for genome editing.

### Exploring the Edit Types Possible with This Inducible CRISPR/Cas9 Tool

The promising [B], [B] + LVA, and [E*](104) CRISPR/Cas9 vectors were further evaluated for their ability to perform other types of genomic edits in S6803.

**Figure 3:**
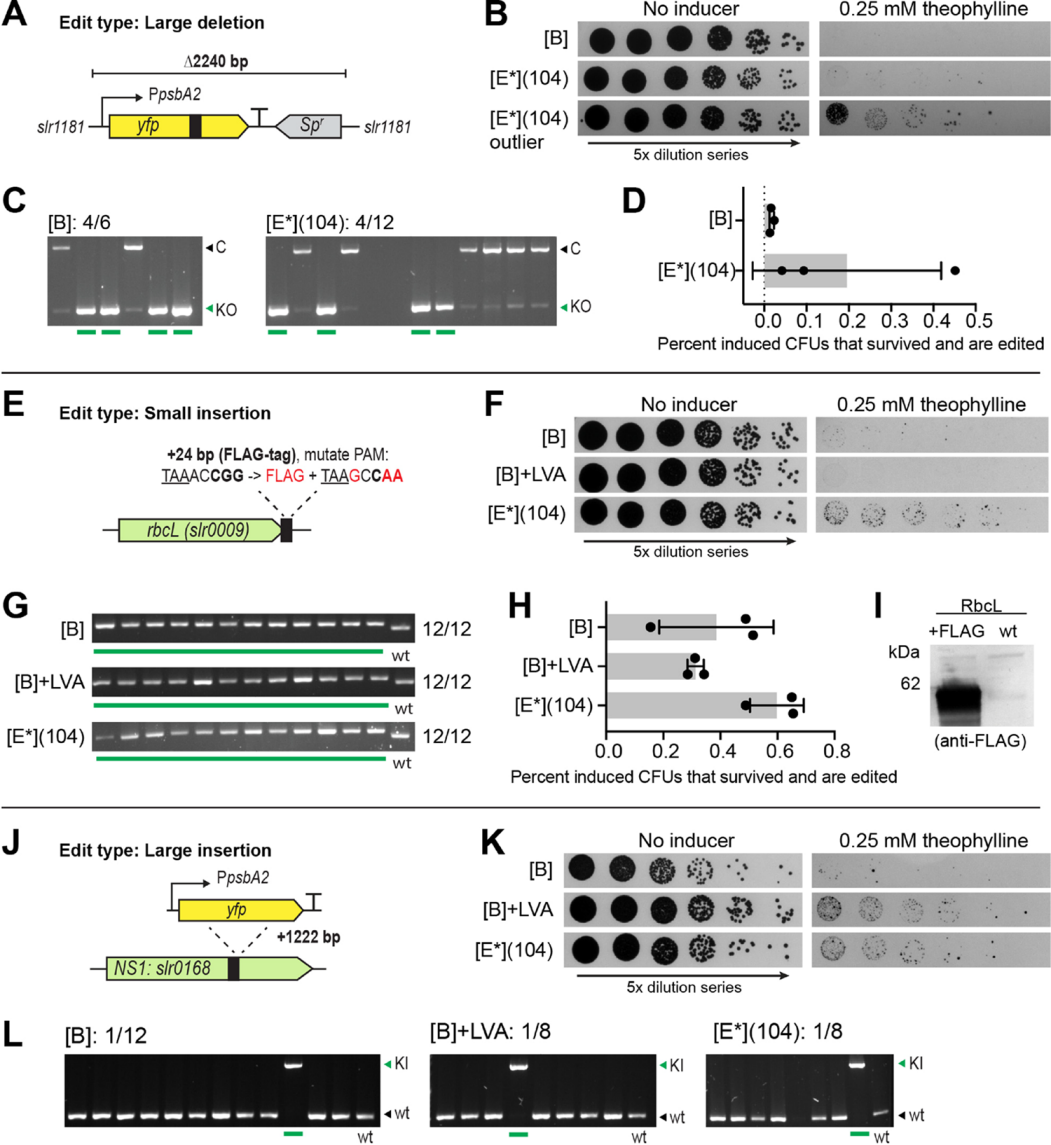
Testing various CRISPR/Cas9-edit types in S6803: (A–D) large deletion of a P_*psbA2*_-Yfp-B0015-Sp^r^ cassette, (E–I) small insertion of a FLAG-tag onto *rbcL*, and (J–L) large insertion of a P_*pbsA2*_-Yfp-B0015 cassette. (A, E, J) Schematics of the different targets in S6803, showing approximate placement of the sgRNA target region (black box) and intended edits. For (E), the TAA stop codon is underlined, the CGG-PAM shown in bold. Mutations introduced in the edit are shown in red. (B, F, K) Induction spot assay results for specified CRISPR/Cas9 constructs. 5× dilution series were plated on plates without or with 0.25 mM theophylline. Done for biological triplicates, representative data is shown. (C, G, L) Editing results for specified CRISPR/Cas9 constructs. A green line below a lane signals a fully edited mutant. Fractions indicated the number of fully edited colonies out of the total number screened. For (C) “C” and “KO” indicate the band size for an unedited and edited colony, respectively. For (L), “wt” and “KI” indicate the band size for an unedited and edited colony, respectively. (D, H) Percentage of induced CFUs that survived (i.e., retained a healthy phenotype) and became edited. Bars show the averages ± SD from biological triplicates, individual values are also shown. (I) Western blot of a fully edited mutant expressing RbcL-FLAG, compared to a wt control; 15 μg protein from the soluble fraction was loaded and probed using an anti-FLAG IgG.

To perform a large deletion, the entire P_*psbA2*_-Yfp-B0015-Sp^r^ cassette (Δ2240 bp) in the Δ*slr1181*::P_*psbA2*_-Yfp-B0015-Sp^r^ strain was targeted (Figure 3a). The same *yfp*-targeting sgRNA as used previously for the small 20 bp deletion was reused here. Although this sgRNA binds in the middle of *yfp* (Figure 3a), far from the edit site, such targeting has been shown to work for CRISPR/Cpf1 deletions in *Anabaena*.^18^ The donor DNA was changed to feature 350 bp homology arms on either side of the *slr1181* integration site. Transformation of the [B], [B] + LVA, and [E*](104) *yfp*-targeting vectors into S6803 resulted in many transformants (Figure S5). Induction of the CRISPR/Cas9 gave few surviving colonies (Figure 3b), the weakest [B] + LVA construct yielded only bleaching colonies (data not shown) and was not considered further for this target. For induced [B] and [E*](104) transformants, the editing efficiencies were 67 and 33%, respectively (Figure 3c). Despite the higher editing efficiency for construct [B], the total percentage of induced CFUs that survived and became edited was higher for the stronger construct [E*](104) (Figure 3d). One outlier for [E*](104) was especially successful.

Next, a small genomic insertion was tested. The aim was to add a C-terminal FLAG-tag (24 bp) to the large subunit of Rubisco (*rbcL*) (Figure 3e). Many proteins of interest lack available antibodies, so the ability to introduce a markerless tag that does not disrupt the region surrounding the gene is a valuable tool. The position of this edit needed to be more specific than the previous deletions, as the FLAG-tag must be added in-frame in front of the *rbcL* stop codon (TAA). The protospacer options in this region were evaluated, and an sgRNA was designed to introduce the DSB two nucleotides from the desired edit site. As the CGG-PAM and parts of the proximal protospacer could not be deleted in this instance, they were instead mutated to stop the edited target from being recognized by the Cas9-sgRNA complex (Figure 3e). Transformation of the [B], [B] + LVA, and [E*](104) *rbcL*-target vectors into S6803 resulted in many transformants (Figure S6a). For constructs [B] + LVA and [E*](104), colonies that survived on the inducer plates directly after transformation were already fully edited (Figure S6b). Inducing CRISPR/Cas9 in the transformants gave few surviving colonies (Figure 3f) but enough so the healthy colony phenotype could be found for all three tested vectors (Figure S6c). The editing efficiency was found to be 100% for all three *rbcL*-targeting constructs (Figure 3g). The higher editing efficiency supported for this edit, compared to the *yfp* one mentioned previously, is likely due to a higher on-target efficiency of the *rbcL*-targeting sgRNA. Despite this, the total percentage of induced CFUs that survived and became edited was not much higher (Figure 3h). The soluble protein fraction from one of the edited colonies was subjected to immunoblotting using an anti-FLAG IgG. Here, the product of the edited *rbcL*, i.e., the RbcL-FLAG, could be clearly detected (Figure 3i).

As a final test, an attempt was made to insert the whole P_*psbA2*_-Yfp-B0015 cassette into neutral site *slr0168* (Figure 3j). This cassette lacked an antibiotic resistance marker, meaning that selection was done only by colonies surviving the Cas9-induced DSBs. The sgRNA was designed to target *slr0168*, and the donor DNA was supplied as three separate pieces when assembling the target vector: one piece for the whole P_*psbA2*_-Yfp-B0015-cassette and one piece each for the upstream and downstream homologous regions (350 bp each). The donor DNA was also designed to remove the TGG-PAM and preceding three bases of the protospacer. Transformation of the [B], [B] + LVA, and [E*](104) target vectors into S6803 resulted in transformants (Figure S7) but fewer than those seen for other target vectors previously. Induction of CRISPR/Cas9 gave many surviving colonies that exhibited the healthier phenotype (Figure 3k). However, editing efficiencies among the screened colonies was low (Figure 3l), as only a single fully edited colony (+1222 bp) was found per construct. It has been shown in *E. coli* that CRISPR/Cas9 editing efficiency is negatively correlated with an increase in insertion length,^45^ likely explaining the lower efficiency seen for this Yfp cassette insertion. This efficiency could possibly be improved by extending the length of the homology arms in the donor DNA;^45^ however, this was not explored here.

### Inducible Multiplexed CRISPR/Cas9 Editing in S6803

Multiplexed, simultaneous editing capabilities could greatly accelerate strain engineering in S6803. Considering the higher editing efficiencies of the smaller edits described above, these were selected for multiplexing tests. Such small edits can be used to modify promoter regions to alter gene expressions or to introduce premature stop codons to knock-out genes.

**Figure 4:**
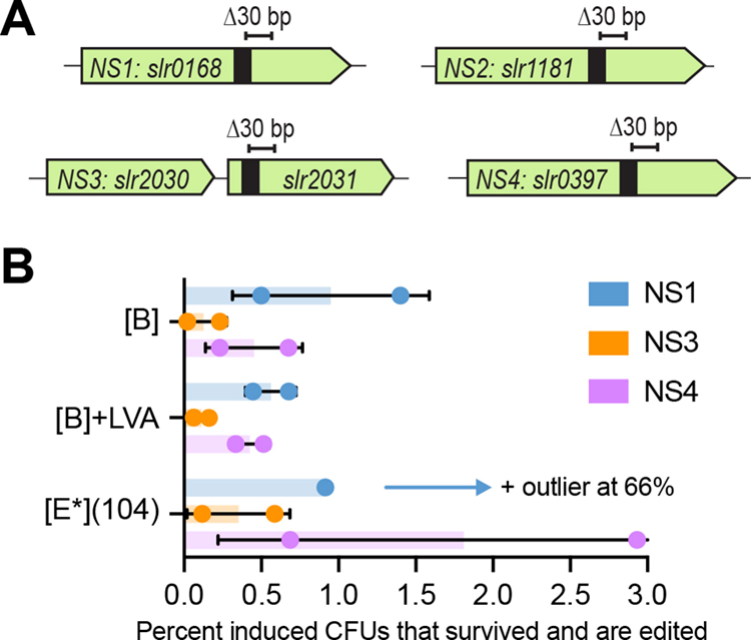
(A) Schematics of the S6803 neutral site (1–4) targets, showing approximate placement of the sgRNA target region (black box) and intended edit (Δ30 bp). (B) Percentage of induced CFUs that survived (retained a healthy phenotype) and became edited, for the individual editing of the NS1, NS3, and NS4 targets. Bars show the averages ± SD from biological duplicates, individual values are also shown. An outlier for [E*](104), target NS1, is not included in the graph but indicated by an arrow.

As a proof-of-principle study, the preliminary goal was to target four neutral sites (NSs) in the S6803 genome and introduce small deletions (Δ30 bp) into all of them with a single pPMQAK1-CRISPR/Cas9 target vector. The NSs were NS1 (*slr0168*), NS2 (*slr1181*), NS3 (*slr2030*–*2031*), and NS4 (*slr0397*); see Figure 4a for the respective sgRNA target regions. The donor DNAs had 350 bp homology arms; the 30 bp deletions included removal of the PAM sites and preceding three bases of the protospacers.

The sgRNA and donor DNA for each of the four targets was first tested individually with the [B], [B] + LVA, and [E*](104) base vectors. Many transformants were obtained for most constructs, with the exception of the NS1- or NS4-targeting [E*](104) constructs (Figure S8). Likely the sgRNAs designed for these two targets are highly efficient, exacerbating the effect of any leaky Cas9 expression from the stronger [E*](104) construct. Induction of CRISPR/Cas9 in these single-target transformants gave varying results depending on the target (Figure S9). When calculating the percentage of induced CFUs that survived and became edited, the best results were seen for targets NS1 and NS4, followed by NS3 (Figure 4b). For NS2, no edited colonies were found (Figure S9d), removing this target from further consideration.

**Figure 5:**
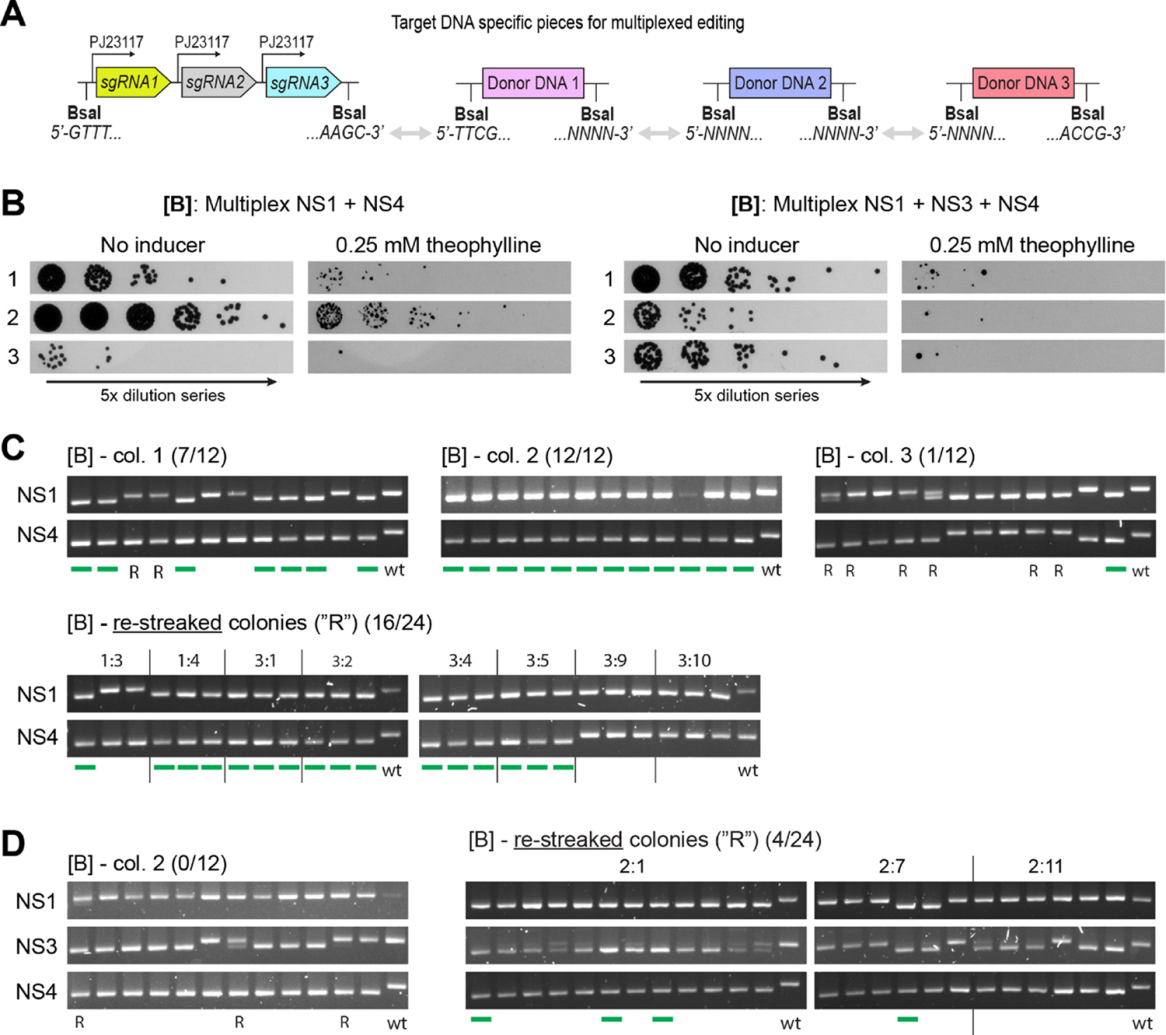
Inducible multiplexed CRISPR/Cas9 editing in S6803. (A) Schematic of the sgRNA and donor DNA pieces built for constructing a multitarget pPMQAK1-CRISPR/Cas9 target vector. The sgRNAs are supplied as a one-piece array, with the same overhangs as a single sgRNA. The donor DNA pieces are supplied as separate parts, with unique overhangs between the donors (*NNNN*), and the standard overhangs on the outermost ends. (B) Induction spot assay results for triplicate transformants of the [B] double- and triple-target constructs. 5× dilution series were plated on plates with or without 0.25 mM theophylline. (C) Editing results for the induced [B] double-target (NS1 + NS4) construct. (D) Editing results for the induced [B] triple-target (NS1 + NS3 + NS4) construct. (C, D) A green line below a lane signals a fully segregated multiedit in that screened colony. Colonies that appear segregated but have not been marked as such are due to them having detectable wt bands when the gels are more closely inspected. Fractions indicated the number of fully edited colonies out of the total number screened. A wt control shows how an unedited colony will appear. An “R” below a lane indicates a not fully segregated mutant that was restreaked for a second round of induction.

Based on these results, it was decided to combine targets NS1 and NS4 in a double-edit target vector, and add the less efficient, but still functional NS3 for a triple-edit target vector. To construct the multiplex target vectors, first the individual sgRNAs were combined by Golden Gate assembly to form a single sgRNA array, which was then combined with the separate donor DNA pieces for simultaneous ligation into the pPMQAK1-CRISPR/Cas9 base vector (Figure 5a). Again, the [B], [B] + LVA, and [E*](104) base vectors were all tested.

Transformation of the multiplex target vectors into S6803 generally resulted in fewer transformants than that seen previously for the single-target vectors (Figure S10). The number of colonies was negatively correlated with the number of targets and expression strength of the CRISPR/Cas9 construct. Still, enough transformants were available that all constructs could be tested.

In a first test, precultures of the transformants were prepared prior to induction. However, this precultivation step appeared to select for cells that had mutated the CRISPR/Cas9 machinery, as most induced cells grew normally on theophylline and very few edits were found in the screened colonies (data not shown). A second attempt was done where the transformants were picked and plated directly on inducer plates (see the Methods section). The representative spot assays now showed more promising results, with fewer cells surviving on the theophylline plates (Figures 5b and S11a). They also showed overall fewer surviving colonies for the triple-target (NS1 + NS3 + NS4) constructs than the double-target (NS1 + NS4) ones.

The induced transformants expressing the [B] (NS1 + NS4) construct showed a large variation in editing efficiency (Figure 5c). For fully segregated colonies, the double-target editing efficiency ranged from 8 to 100%. Several screened colonies were edited only in one target or showed incomplete segregation; some of these latter ones could be pushed toward full segregation by restreaking on new inducer plates. It is likely that the segregation-resistant colonies had escaped the action of CRISPR/Cas9 by mutating it, thereby remaining unedited but still viable. For the [B] + LVA (NS1 + NS4) construct, restreaking incompletely segregated colonies in a second round of induction ultimately yielded some double mutants (Figure S11b). However, for the [E*](104) (NS1 + NS4) construct, while all screened colonies were fully segregated at the NS4-target (Figure S11b), there was no trace of editing in the NS1 target and a second round of induction was not attempted.

For the [B] (NS1 + NS3 + NS4) construct, only after a second round of induction were a few fully segregated triple mutants identified (Figure 5d). Several more colonies were almost but not yet fully segregated. The poor segregation of edits was observed mainly at the NS3-target site, which was known from the single-target data to be less amenable to editing than NS1 or NS4.

For the [B] + LVA (NS1 + NS3 + NS4) construct, edits were generally rare in any of the targets (Figure S11c); likely this weakest CRISPR/Cas9 construct was too weak to support this multiediting attempt. For the [E*](104) (NS1 + NS3 + NS4) construct, the high survival on theophylline (Figure S11a) combined with a few edits (Figure S11c) indicated that these had likely mutated the CRISPR/Cas9. Possibly the CRISPR/Cas9 activity for these [E*](104) constructs was too strong, causing cells to die from too rapidly occurring multiple DSBs or having already “escaped” the CRISPR/Cas9 by selecting against the effects of a leaky *cas9*-expression.

The different multiplexing successes for the varyingly strong CRISPR/Cas9 constructs highlight the usefulness in having these options. Overall, the medium-strength construct [B] performed the best. Taken together, these results showed that multiplexed CRISPR/Cas9 editing using one single-target vector is possible in S6803.

### Evaluating a Nickel-Inducible Curing System To Clear Mutants of the CRISPR/Cas9 Vector

A desirable feature of a CRISPR/Cas9 vector is that it should be easily cleared from cells after editing, leaving the mutant free of antibiotic resistance markers. A common curing method is to grow edited cells without selecting for the vector, plate this, and screen colonies for plasmid loss. This method would likely be inefficient for the CRISPR/Cas9 vectors developed in this study, as RSF1010-replicon vectors are stably maintained in S6803 for prolonged periods under nonselective conditions.^46^ Another option is to add a counter-selection marker to the vector, such as *sacB* from *Bacillus subtilis* that causes sucrose sensitivity.^47^ However, this *sacB* method is only functional for the glucose-tolerant subset of wild-type S6803 strains.^48,49^ An alternative counter-selection system developed to work in all wild-type strains of S6803 is based on the endoribonuclease-encoding *mazF* from *E. coli* that causes protein synthesis inhibition and ultimately cell death.^48^ This *mazF* was here tested for its ability to drive inducible curing of the pPMQAK1-CRISPR/Cas9 vector. A Ni^2+^-inducible promoter was used to drive *mazF* expression, making its expression separately controlled from the CRISPR/Cas9 vector. Two Ni^2+^-inducible promoters, both native to S6803, were compared to identify the most suitable. P_*nrsB*_ drives expression of the Ni^2+^-resistance operon (*nrsBACD*) and is controlled by the Ni^2+^-sensing NrsS and transcriptional regulator NrsR.^50^ P_*nrsD*_ drives only *nrsD* expression and is controlled by the InrS transcriptional regulator.^51^ As both promoters are native to S6803, the regulators are encoded from its own genome. To reduce the risk of premature toxicity from leaky *mazF* expression, it was combined with either of two *ssrA* protease degradation tags: one stronger (LVA) and one weaker (AAV).^41^ This gave four different combinations of the *mazF*-curing system to test.

**Figure 6:**
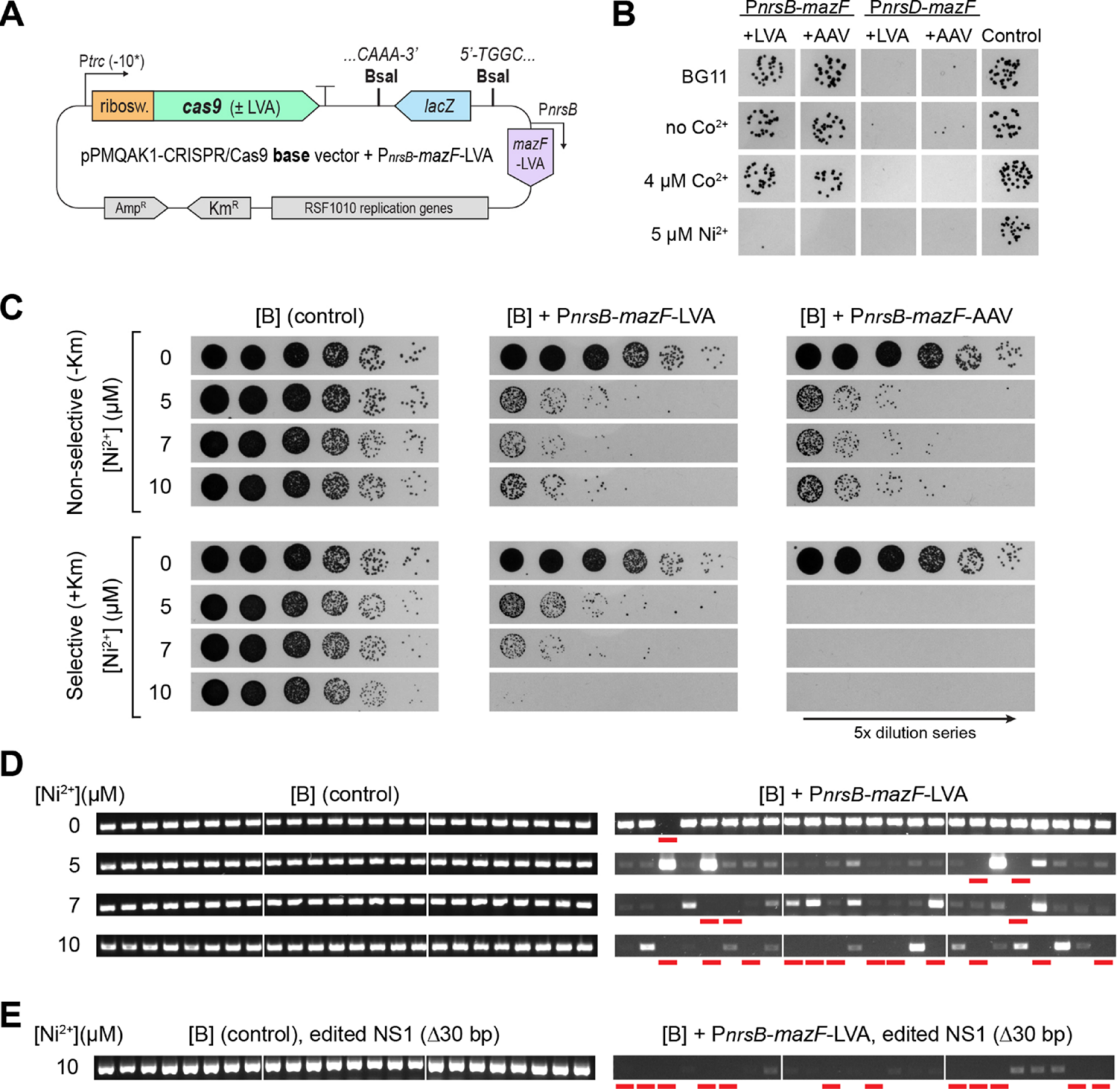
Evaluating a Ni^2+^-inducible curing system for the pPMQAK1-CRISPR/Cas9 vector. (A) Schematic showing the pPMQAK1-CRISPR/Cas9 base vector supplemented with the best performing curing system: P_*nrsB*_-*mazF*-LVA. The other evaluated curing systems featured either the P_*nrsB*_- or the P_*nrsD*_-promoter, and either an LVA- or an AAV-tag added to *mazF*. (B) Transformation results for [B] base vectors supplemented with the indicated curing system. A control [B] base vector without a curing system was included. Transformed cells were plated on regular BG11-plates, plates without any Co^2+^, or ones with 4 μM Co^2+^ or 5 μM Ni^2+^. (C) Ni^2+^-induction spot assay results for S6803 containing the [B] base vectors, either supplemented with P_*nrsB*_-*mazF*-LVA or P_*nrsB*_-*mazF*-AAV, or a control lacking a curing system. 5× dilution series were plated on nonselective (no kanamycin) or selective plates supplemented with 0, 5, 7, and 10 μM Ni^2+^. Done for biological triplicates, representative data are shown. (D) Resulting colonies after Ni^2+^-induction were screened for curing of the pPMQAK1-CRISPR/Cas9 vector. Screening was done of the *kmR*-gene found on the pPMQAK1-backbone. Screening results for the Ni^2+^-induced [B] (control) or [B] + P_*nrsB*_-*mazF*-LVA base vectors are shown. For each induced biological triplicate, eight colonies were screened. A red line below a lane signals a fully cured mutant. Colonies that appear cured but have not been marked as such is due to them having detectable traces of a pPMQAK1-originating band when gels are more closely inspected. (E) Triplicate NS1-edited, fully segregated mutants induced on nonselective BG11-plates with 10 μM Ni^2+^ were screened for pPMQAK1-vector curing. For each induced biological triplicate, eight colonies were screened. A red line below a lane signals a fully cured mutant. Colonies that appear cured but have not been marked as such are due to them having detectable traces of a pPMQAK1-originating band when gels are more closely inspected.

The four curing system combinations were evaluated in S6803 by adding them onto the [B]-variant CRISPR/Cas9 base vector that has *lacZ* in place of an sgRNA and donor DNA (Figures 6a and S3b). When electroporated into S6803, the vectors with either P_*nrsD*_-*mazF* system resulted in few surviving transformants (Figure 6b), likely due to a lethal level of leaky *mazF* expression. The vectors with the P_*nrsB*_-*mazF* variants yielded colonies in a comparable number to the control vector lacking any curing system (Figure 6b). No clear difference could be observed between the vectors with P_*nrsB*_-*mazF* combined with either the LVA-tag or the AAV-tag. On plates supplemented with 5 μM Ni^2+^, there were no or few transformants for either of the P_*nrsB*_-*mazF* vectors (Figure 6b), indicating that induction of the toxic *mazF* was successful. Despite P_*nrsB*_ being reported to be both Ni^2+^- and Co^2+^-inducible,^51,52^ induction by Co^2+^ appeared negligible as plating on regular BG11 (containing 0.17 μM Co^2+^), Co^2+^-free BG11, or plates with 4 μM Co^2+^ produced similar results (Figure 6b).

To determine reliable curing conditions, S6803 transformed with either of the P_*nrsB*_-*mazF*-supplemented [B] base vectors were plated on BG11-plates with 5, 7, or 10 μM Ni^2+^. Ni^2+^-induced expression of the toxic *mazF* by either of the tested curing systems caused cell death for a majority of the cells (Figure 6c). Nonselective plates (no kanamycin) were used to allow for cured colonies to grow, while selective plates were included to assess the frequency of colonies that managed to escape the *mazF* counter-selection. Cells containing the vector with P_*nrsB*_-*mazF*-LVA showed a distinct difference in the number of background colonies on selective Ni^2+^-plates, where the highest 10 μM Ni^2+^ gave the lowest level of background (Figure 6c). Cells containing the vector with P_*nrsB*_-*mazF*-AAV showed no background already at 5 μM Ni^2+^, in line with the AAV-tag being a weaker degradation tag than LVA and supporting higher *mazF* levels at lower Ni^2+^ concentrations. It was decided that the weaker but titratable LVA system would be more suitable for use with the CRISPR/Cas9 vectors. Colonies that grew on Ni^2+^-supplemented, nonselective plates were screened by colony-PCR to determine if they had been cured, i.e., if all traces of the pPMQAK1-vector were gone. The results showed that without Ni^2+^, the stability of the vector with P_*nrsB*_-*mazF*-LVA was comparable to the control vector lacking a curing system, indicating tight control of the curing system (Figure 6d). When exposed to Ni^2+^, the stability of the control vector was unaffected while the cellular amount of the vector with P_*nrsB*_-*mazF*-LVA was dramatically reduced (Figure 6d). The curing was most efficient at 10 μM Ni^2+^. Depending on the biological replicate, 37.5–75% of screened colonies were cured, a significant improvement over the 0% for the control.

To ensure that addition of P_*nrsB*_-*mazF*-LVA to the [B] CRISPR/Cas9 vector did not have a negative impact on gene editing, its editing ability was compared to a control vector lacking a curing system. Several previously tested edit targets were revisited, and the results showed that the moderate editing efficiency of *yfp* (Δ20 bp), the high editing efficiency of NS1 (Δ30 bp), and multiplexed editing of NS1 + NS4 (Δ30 bp) were all maintained with the new P_*nrsB*_-*mazF*-LVA supplemented [B] vector (Figure S12b–d). The number of obtained transformants was also comparable (Figure S12a). Additionally, Ni^2+^-induction of NS1-edited cells resulted in vector curing in 25–62.5% of screened colonies, showing that the curing system remains intact following a CRISPR/Cas9 edit step (Figure 6e). These results are expected to also apply to the [B] + LVA and [E*](104) vector variants, as the curing is independent of the CRISPR/Cas9 and induced separately.

### Assessing the Final CRISPR/Cas9 System by Targeting the Commonly Tested *nblA* Deletion Target

**Figure 7:**
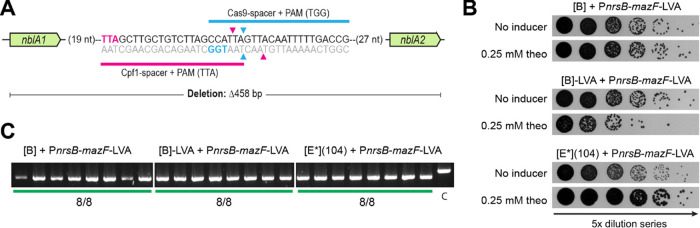
Evaluating the deletion (Δ458 bp) of *nblA1–2* using the P_*nrsB*_-*mazF*-LVA supplemented CRISPR/Cas9 vectors. (A) Schematic of the region between *nblA1* and *nblA2* in S6803, showing the relative location of target regions used with Cas9 (this study) and with Cpf1 (previously published, see main text for references). Marked in color in the sequence region are the PAMs for either the Cas9 (blue) or Cpf1 (pink) target regions. The arrowheads indicate where the Cas9 (blue) or Cpf1 (pink) will introduce DSBs when targeted to the indicated regions; Cas9 produces blunt DSBs while Cpf1 produces staggered DSBs. (B) Induction spot assay results for [B], [B] + LVA, and [E*](104), all supplemented with P_*nrsB*_-*mazF*-LVA. 5× dilution series were plated on regular BG11-plates or ones supplemented with 0.25 mM theophylline inducer. Done for biological triplicates, representative data is shown. (C) Editing results of the *nblA1–2* deletion target for [B], [B] + LVA, and [E*](104), all supplemented with P_*nrsB*_-*mazF*-LVA. A green line below a lane signals a fully edited (Δ458 bp) mutant. A control (“C”) shows how an unedited colony will appear. Fractions indicated the number of fully edited colonies out of the total number screened.

Lastly, we aimed to compare the inducible, curable CRISPR/Cas9 system described in this study to a plasmid-delivered Cpf1-based CRISPR system that has been characterized in S6803 previously.^17,53^ A direct comparison is difficult due to the different PAM requirements and therefore protospacer selection for these two endonucleases.^9^ For an indirect comparison, we targeted the two genes *nblA1–2* (nonbleaching protein A) for deletion using a Cas9-compatible spacer that targeted as close as possible to the previously evaluated Cpf1-compatible target site (Figure 7a). Due to the different cleavage mechanisms of Cas9 and Cpf1,^12,54^ the designed sgRNA directed the Cas9 to make a blunt DSB in the same area, as the Cpf1 had previously been directed to make a staggered cut. Unlike previously tested sgRNAs in this study, this one targeted the nontemplate strand of the target. The same donor DNA as specified in the Cpf1-studies, with 1 kb long homology arms,^17,53^ was reused here. The editing was attempted with all three P_*nrsB*_-*mazF*-LVA-supplemented CRISPR/Cas9 vectors, i.e., [B], [B] + LVA, and [E*](104). Upon electroporation, obtained colonies (Figure S13a) were induced for CRISPR/Cas9 editing on 0.25 mM theophylline. Unexpectedly, most cells survived this induction (Figure 7b) and all screened colonies, independent of the CRISPR/Cas9 vector, were found to have the fully segregated *nblA1–2* (Δ458 bp) deletion (Figure 7c). This high editing efficiency was likely due to leaky CRISPR/Cas9-editing occurring prior to induction. This hypothesis was confirmed when uninduced colonies from the transformation plates were screened, the progression of leaky editing was found to correspond to the potential strength of the CRISPR/Cas9 vector (Figure S13b). It is also possible that the use of a sgRNA targeting the nontemplate strand, known for resulting in higher on-target efficiencies,^44^ contributed to the observed leaky editing. Despite this, the fast segregation of this target in 100% of screened colonies stands in contrast to the three induction rounds required to achieve segregated Δ*nblA1–2* with Cpf1.^17,53^ Therefore, the described CRISPR/Cas9 system has the potential advantage of reducing the time that is needed to obtain segregated mutants, despite utilizing a two-step process where transformation and induced CRISPR/Cas9 editing are done separately.

## Conclusions

In this study, we designed a riboswitch-based, theophylline-inducible CRISPR/Cas9-system for easy use in S6803. This system forgoes the common issue of Cas9-toxicity, enabling one to attain high transformation efficiencies for the pPMQAK1-CRISPR/Cas9 target vector. Inducing CRISPR/Cas9 in obtained transformants allowed edited colonies to be isolated. Single edits were fully segregated after just one passage on the inducer, reducing the editing time. The system was also shown to support multiplexed editing in S6803, enabling simultaneous editing of up to three targets using one single CRISPR/Cas9 vector. The [B] construct was most successful overall, supporting all attempted single and multiplexed edits. Although, the stronger [E*](104) and weaker [B] + LVA constructs could be useful in the case of weak or strong sgRNAs, respectively, that cannot be redesigned due to, e.g., specific target site requirements. The two-step workflow, where transformation and CRISPR/Cas9 genome editing are done separately, was most reliable throughout this study. However, in some cases (e.g., adding the FLAG-tag to *rbcL*), edited colonies could be found among transformants that survived induction directly after transformation. Finally, the developed CRISPR/Cas9 vectors were equipped with a curing system based on the nickel-inducible expression of the toxic *mazF*. The combination of P_*nrsB*_ and an LVA-tag on the *mazF* allowed for a tightly controlled curing system that did not interfere with CRISPR/Cas9 editing and that supported curing in 25–75% of screened cells.

## Methods

### Strains and General Growth Conditions

See Table S1 for strains used in this study. The wild-type S6803 (a gift from Martin Fulda, University of Goettingen) is a nonmotile GT-S derivative. A Δ*slr1181*::P_*psbA2*_-Yfp-B0015-Sp^r^ strain was used for *yfp* genome editing. All cultivations were done at 30 °C, 1% (v/v) CO_2_, and 30 (plates) or 50 (liquid) μE s^–1^ m^2^, using a Percival Climatics SE-1100 climate chamber. The BG11 media was buffered to pH 7.9 with 25 mM HEPES. For growth on solid media, 1.5% (w/v) agar and 0.3% (w/v) sodium thiosulfate were added to the BG11. When needed, antibiotics were supplemented (40 μg/mL kanamycin, 40 μg/mL spectinomycin). Growth in liquid media was monitored by OD_730_. Special conditions for, e.g., CRISPR/Cas9-induction or plasmid curing are explained in the following sections.

### Vector Construction

For vectors used and constructed in this study, see Table S1. All the primers are listed in Table S2. All subcloning was done in *Escherichia coli* XL1-Blue.

The pPMQAK1-T vector^40^ has BpiI sites ready for Golden Gate cloning.^55^ The Gfp reporter, and CRISPR/Cas9 base vectors were built by amplifying the required inserts, which added overhangs with compatible BpiI sites, and performing multipiece assembly into pPMQAK1-T (see more details as follows). All Golden Gate cloning was done using Thermo Fisher Scientific T4 DNA ligase and FastDigest BpiI or BsaI (as specified). All Golden Gate cloning primers were designed using Benchling.^56^

The sequences of the evaluated promoters, P_*conII*_ and P_*trc*_, and riboswitches (B, C, E*) can be seen in the Supporting Information. The combinations were ordered as “Ultramer”-oligos from IDT.

To build the Gfp reporter plasmids, the *gfp* and terminator (BBa_B0015) was amplified as a single piece from a plasmid available in lab. The promoter-riboswitch pieces were amplified from the “Ultramer” templates.

Before constructing the CRISPR/Cas9 base vectors, the pPMQAK1-T vector and *cas9* gene were domesticated for use with BsaI and BpiI, respectively. This was done either by PCR-based site-directed mutagenesis^57^ or subcloning using Golden Gate assembly. To build the CRISPR/Cas9 base vectors, the following pieces were amplified and inserted into pPMQAK1-T: the P_*trc*_ riboswitch pieces using the “Ultramer” templates, the BpiI-free *cas9* gene, a BBa_B0015 terminator, and the *lacZ* gene as found in the pPMQAK1-T with extra added BsaI sites on either side for future cloning use. For the promoterless-*cas9* control vector, the P_*trc*_ riboswitch piece was left out. The constructed pPMQAK1-CRISPR/Cas9 base vectors, with P_*trc*_ riboswitch (B, C, or E*), were used in site-directed mutagenesis^57^ to make variants with an LVA-tag on Cas9, and variants with a weaker −10-box in P_*trc*_.

For the P_*nrsB*_-*mazF*-LVA/AAV or P_*nrsD*_-*mazF*-LVA/AAV supplemented CRISPR/Cas9 base vectors, the promoters (see the Supporting Information for sequences) and *mazF* (with added LVA- or AAV-tag) were added as extra pieces in the base vector assembly described above. P_*nrsB*_ and P_*nrsD*_ were amplified from the S6803 genome, while *mazF* was amplified from *E. coli* cells.

The pPMQAK1-CRISPR/Cas9 target vectors were built by Golden Gate cloning; the sgRNA and donor DNA were simultaneously inserted using the BsaI sites in the selected CRISPR/Cas9 base vector. The BsaI-containing overhangs added to the 5′- and 3′-ends of the amplified sgRNA and donor DNA were kept constant, see Supporting Information for details.

The single-target sgRNAs were constructed by overlap-PCR, using the compatible spacer sequences added as overhangs to the BBa_J23117 promoter piece and the Cas9-handle-*S. pyogenes*-terminator piece. An example sgRNA sequence can be seen in the Supporting Information. For the multiplex target vectors, the sgRNAs were first combined into an array by Golden Gate subcloning in a pMD19-backbone. This sgRNA array construction was done as described by Li et al.^45^ The final pMD19-sgRNA array plasmid contained the necessary BsaI sites flanking the array, so it was directly added to the target vector Golden Gate assembly. The sgRNA spacers in this study (Table S3) were designed using Benchling.^56^ Off-target binding analysis (Table S4) was done using Benchling^56^ and the CasOT software.^58^

The donor DNAs were prepared by overlap-PCR of the amplified homology arms. An exception was when inserting the P_*psbA2*_-Yfp-B0015 cassette into *slr0168*; here, the two homology arms and cassette were supplied as three separate pieces. For the multiplex target vectors, the donor DNA for each site was prepared separately, with overhangs as shown in Figure 5a.

### Strain Construction

All constructed pPMQAK1 vectors were transformed into S6803 by electroporation, see Supporting Information for details. Each transformation used 5–10 mL exponentially growing S6803 (OD_730_ 0.6–1), and 100–350 ng of vector DNA. Cells were recovered for 16–24 h, before plating on selective media. For CRISPR/Cas9 vectors the recovered cells were pelleted, resuspended in 460 μL BG11, whereby 200 μL each was plated on selective BG11-plates without or with 0.25 mM theophylline. For preparation and use of the 200 mM theophylline stock, see the Supporting Information. In some cases (e.g., the *yfp* edit, and testing the *mazF*-supplemented vectors), the remaining cell suspensions were used to make spot assays (10 μL spots).

### Inducing CRISPR/Cas9 Genome Editing

Induction was done on solid media. BG11-plates were supplemented with 0.25 mM (or as indicated) theophylline. Induction was done for biological triplicates (i.e., separate colonies from one transformation) in all cases except for the biological duplicates used for the single-target editing of NS1, 2, 3, or 4. For the *yfp* (Δ20 bp) edit, induction of the biological triplicates was also prepared in technical duplicates. Due to the similar results between these technical duplicates (Figure 2e), such technical replicates were not included for any of the following inductions. Transformants used for inductions always came from freshly prepared transformations (no older than 4 weeks); all strains compared in one induction experiment were always transformed on the same day. Induction was done in one of two ways; transformants were picked into precultures (∼2–3 mL, done in 24-deep well plates) and cultivated for 3–4 days before plated on inducer plates, or transformant colonies were suspended in a small volume (∼50–80 μL) of BG11 and directly plated on inducer plates. The latter method is recommended. The described precultures or colony suspensions were diluted before plating on inducer plates. The prepared “base”-dilution was OD_730_ 0.2, from which a 5× dilution series was prepared. The dilution series were used to make spot assays (4 μL spots) on indicated plate types, and the remaining volume (40–60 μL) of selected dilutions (commonly 5×, 25×, 125×) was then plated on full-size inducer plates, see Supporting Information for details. After 10–14 days, healthy-looking colonies were screened. If needed, colonies with unsegregated edits were restreaked on new inducer plates.

### Evaluating Genome Editing and Screening Mutants

After CRISPR/Cas9-induction, surviving healthy-looking colonies were screened by colony-PCR. The editing efficiency was the percentage of edited colonies found among the ones screened. The second quantification used, the percentage of induced CFUs that survived and also became edited, was calculated by relating the found editing efficiency to the percentage of induced CFUs that retained a healthy phenotype (i.e., “survived”). This latter percentage was estimated from the spot assays prepared for every editing experiment; the total plated CFUs and surviving, healthy-looking CFUs were estimated from the plates without or with theophylline, respectively. Healthy-looking meant colonies that were not bleached, but instead green and had managed to grow well on the inducer plates. Raw data for these CFU calculations can be seen in Table S5.

For the RbcL-FLAG immunoblot, 15 μg protein (soluble fraction) was separated by SDS-PAGE, transferred onto a 0.45 μm PVDF membrane, and analyzed using the WesternBreeze Chromogenic anti-mouse kit with a primary anti-FLAG (F4042, Sigma-Aldrich) antibody at a 4000× dilution. Cells were lysed by bead beating (200 μL beads, 10 min total, 1 min on/off, 4 °C) in lysis buffer (50 Tris–HCl, 150 mM NaCl, Roche complete EDTA-free protease inhibitor); centrifugation for 30 min at 4 °C and 21,000 × *g* allowed isolation of the soluble fraction supernatant.

### Curing of pPMQAK1-CRISPR/Cas9 Vectors

For evaluation of the optimal curing conditions, triplicate colonies were suspended separately in a small volume of BG11 and then plated on selective and nonselective (without kanamycin) BG11-plates supplemented with 0, 5, 7, or 10 μM Ni^2+^ (added using a 50 mM NiSO_4_ stock). Both spot plates (4 μL spots of a 5× dilution series) and spreading on full-sized plates was done. The resulting colonies were screened for pPMQAK1-loss (i.e., curing) by screening for the plasmid-encoded kanamycin-resistance cassette by colony-PCR.

Curing of edited, fully segregated colonies was performed by suspending them in a small volume of BG11 (∼80 μL) and spreading this on nonselective (without kanamycin) BG11-plate supplemented 10 μM Ni^2+^. Screening resulting colonies for pPMQAK1 loss was done as described above.

## Supporting Information Available

Sequences of interest, and a general step-by-step method description for using the CRISPR/Cas9-system; evaluation of the toxicity of theophylline towards S6803; P*_conII_* and P*_trc_* in combination with riboswitches B, C, and E* compared using a Gfp-reporter; detailed vector maps of the designed CRISPR/Cas9 base vectors; additional results for *yfp*-targeting (Δ20 bp) in Δ*slr1181*::P*_psbA2_*-Yfp-B0015-Spr; additional results for *yfp*-targeting (Δ2240 bp, whole Yfp-cassette) in Δ*slr1181*::P*_psbA2_*-Yfp-B0015-Spr; additional results for *rbcL*-targeting (adding a C-terminal FLAG); additional results for *slr0168*-targeting (+1220 bp, P*_psbA2_*-Yfp-B0015 cassette)); additional results for induced editing (Δ30 bp) of NS1-4 as single targets; additional results for induced multi-editing of the combined targets NS1+NS4 and NS1+NS3+NS4; additional results from comparing the editing ability of construct [B] when supplemented with P*_nrsB_*-*mazF*-LVA or without a curing system; additional results for *nblA1-2* targeting; strains and vectors used or constructed in this study; primers used in this study; details for all tested sgRNA-spacers; off-target analysis of sgRNAs used in this study; raw data for the calculated CFUs for selected CRISPR/Cas9 editing experiments (PDF)

## Abbreviations

CRISPR: clustered regularly interspaced short palindromic repeats
Cas: CRISPR-associated
sgRNA: single chimeric guide RNA
PAM: protospacer adjacent motif
DSB: double-stranded break
HDR: homology-directed repair
RBS: ribosome binding site
CFU: colony-forming unit
wt: wild type
NS: neutral site.
