# Supplementary material for: Inducible CRISPR/Cas9 Allows for Multiplexed and Rapidly Segregated Single-Target Genome Editing in Synechocystis Sp. PCC 6803
Source: ACS Synth Biol. 2022 Aug 15;11(9):3100–13. doi: 10.1021/acssynbio.2c00375 (PMC9486961; doi:10.1021/acssynbio.2c00375)
Supplement: Supplementary file 1 — sb2c00375_si_001.pdf [file sb2c00375_si_001.pdf]

Supporting information

Inducible CRISPR/Cas9 allows for multiplexed and rapidly segregated single target genome editing in *Synechocystis* sp. PCC 6803

Ivana Cengic[a], Inés C. Cañadas[b], Nigel P. Minton[b], Elton P. Hudson[a] *

[a]School of Engineering Sciences in Chemistry, Biotechnology and Health, KTH Royal Institute of Technology, Science for Life Laboratory, Stockholm, Sweden

[b]BBSRC/EPSRC Synthetic Biology Research Centre (SBRC), School of Life Sciences, The University of Nottingham, Nottingham, NG7 2RD, United Kingdom

*Corresponding author: huds@kth.se

Contents:

- Figure S1-S13
- Table S1-S5
- Sequences of promoters (P_{conII}, P_{trc}, P_{nrsB}, P_{nrsD}) and riboswitches (B, C, E*)
- Extra methods - Step-by-step protocol of using the CRISPR/Cas9-system

Figures

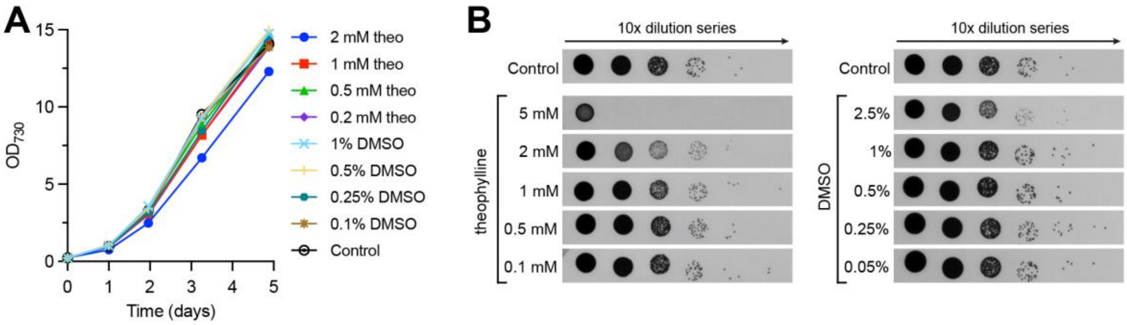

Figure S1. Evaluating the toxicity of theophylline towards S6803. (**A**) Single cultures were grown in BG11 supplemented with various concentrations of theophylline, or the respective amount of DMSO-carrier only. A control with neither was included. (**B**) Spot assay on BG11-plates supplemented with the indicated concentrations of theophylline or DMSO-carrier only. A control with neither was included. A wt S6803 culture was diluted to OD_{730} 0.2 and used to prepare a 10x dilution series that was plated (4 µl spots). Shown is representative data from two separate experiments.

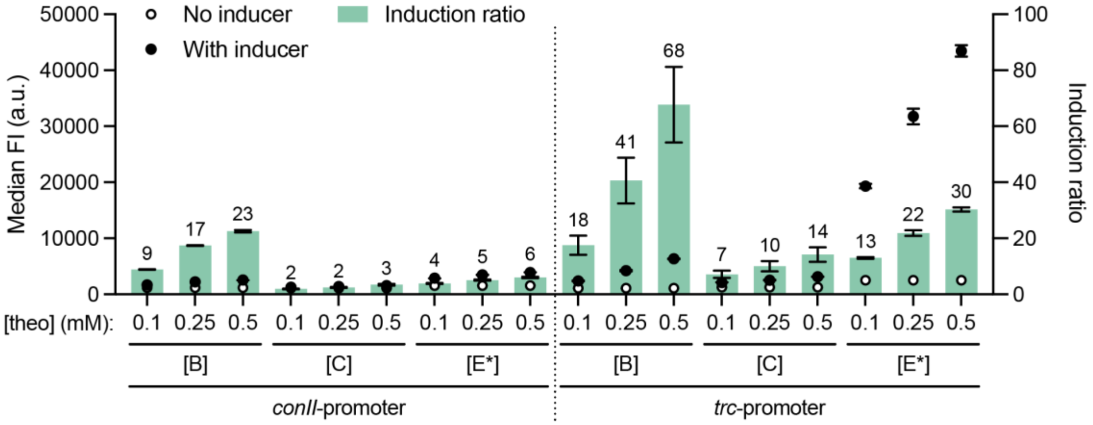

Figure S2. P_{conII} and P_{trc} in combination with riboswitches B, C, and E* were compared using a Gfp-reporter. Three non-toxic theophylline concentrations were tested: 0.1, 0.25, and 0.5 mM. Samples with no added inducer were supplemented with the respective amount of DMSO-carrier: 0.05%, 0.125%, and 0.25%. Fluorescence was measured 3 days after induction by flow cytometry (Beckman Coulter CytoFLEX, FITC-channel: emission 525 nm, excitation 488 nm). 10,000 events were acquired; data analysis was done using FlowJo (FlowJo LLC). The left y-axis shows the average median fluorescence intensity (filled or empty circle); the right y-axis shows the induction ratio (induced signal divided by un-induced signal, both values were first normalized against the wild type signal). The numbers above the bars are the calculated average induction ratios. All data is presented as averages ± SD from biological duplicates. Non-visible error-bars are smaller than the data symbol.

A

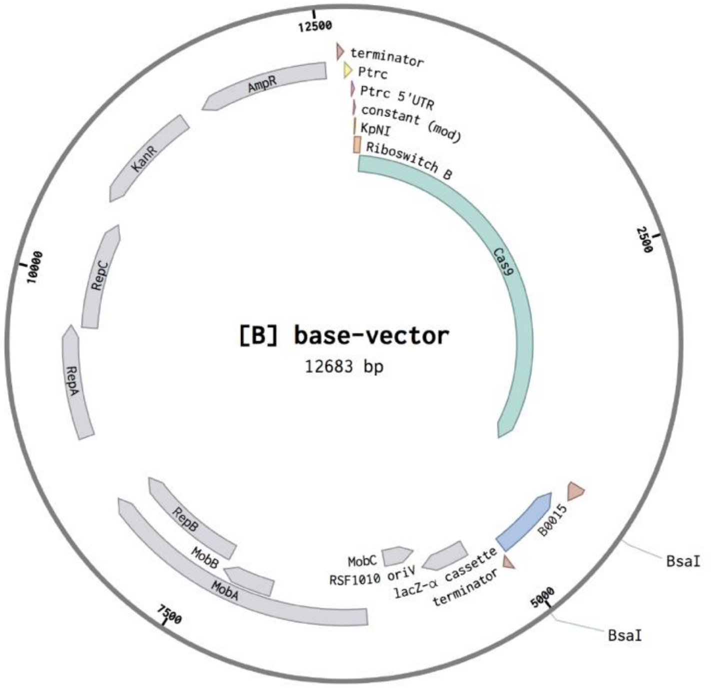

B

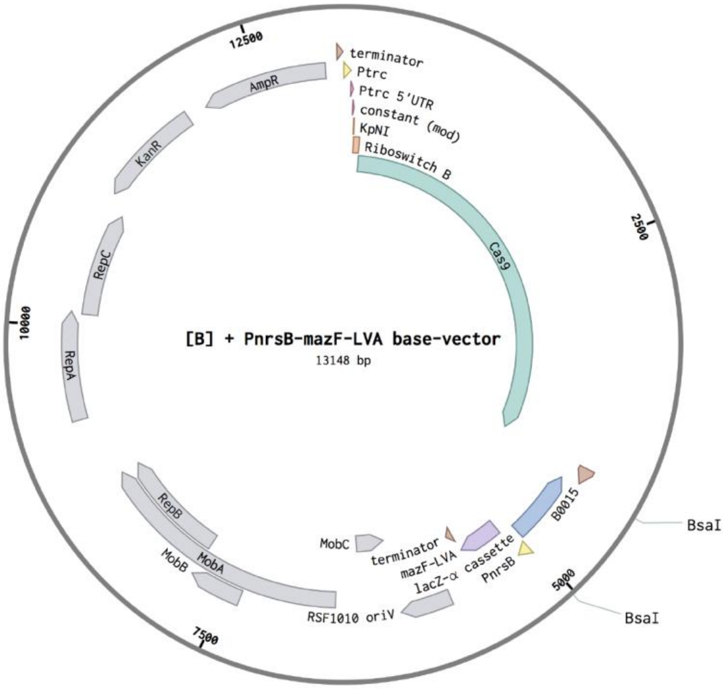

Figure S3. Detailed vector maps of the designed CRISPR/Cas9 base vectors. Only an example vector map for riboswitch-variant [B] is shown as most elements are shared between the vectors; exceptions are the LVA-tag on Cas9 for [B]-LVA, and the riboswitch and -10-box for [E*](104). (**A**) For base vector [B]. (**B**) For base vector [B] supplemented with the P$_{nrsB}$-*mazF*-LVA curing system.

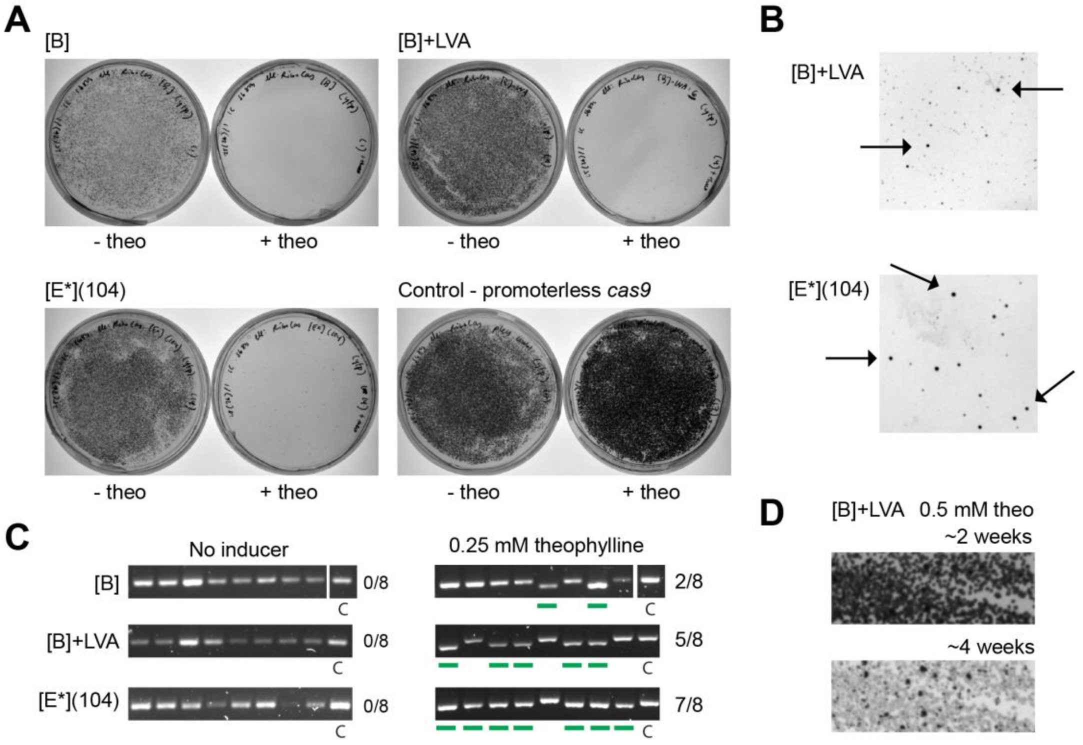

Figure S4. (**A**) Full plates of S6803 Δ*slr1181*::P_{psbA2}-Yfp-B0015-Sp^{r}, transformed with *yfp*-targeting (Δ20 bp) pPMQAK1-CRISPR/Cas9 vector variants [B], [B]+LVA, [E*](104), and the control (no Cas9 expression). Plates without (-theo) and with 0.25 mM theophylline (+theo) are both shown. (**B**) Zoomed in sections of the +theo plates from (A) for construct variants [B]+LVA and [E*](104), showing surviving colonies. Arrows indicate examples of larger colonies, screened for editing. (**C**) Screening colonies from the transformation plates seen in (A), both from ones without and ones with inducer. A green line below a lane signals a fully edited (Δ20 bp) mutant. A control ("C") shows how an unedited colony will appear. Fractions indicated the number of fully edited colonies out of the total number screened. (**D**) Appearance of cells expressing the *yfp*-targeting (Δ20 bp) [B]+LVA vector, after 2 and 4 weeks on 0.5 mM theophylline inducer.

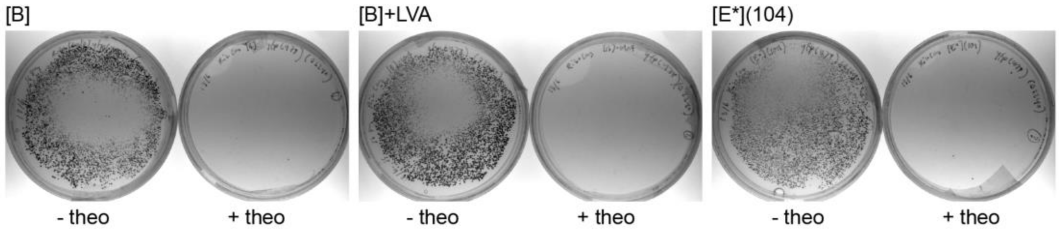

Figure S5. Full plates of S6803 Δ*slr1181*::P_{psbA2}-Yfp-B0015-Sp^{r} transformed with *yfp*-targeting (Δ2240 bp, whole Yfp-cassette) pPMQAK1-CRISPR/Cas9 vector variants [B], [B]+LVA, and [E*](104). Selective plates without (-theo) and with 0.25 mM theophylline (+theo) are both shown.

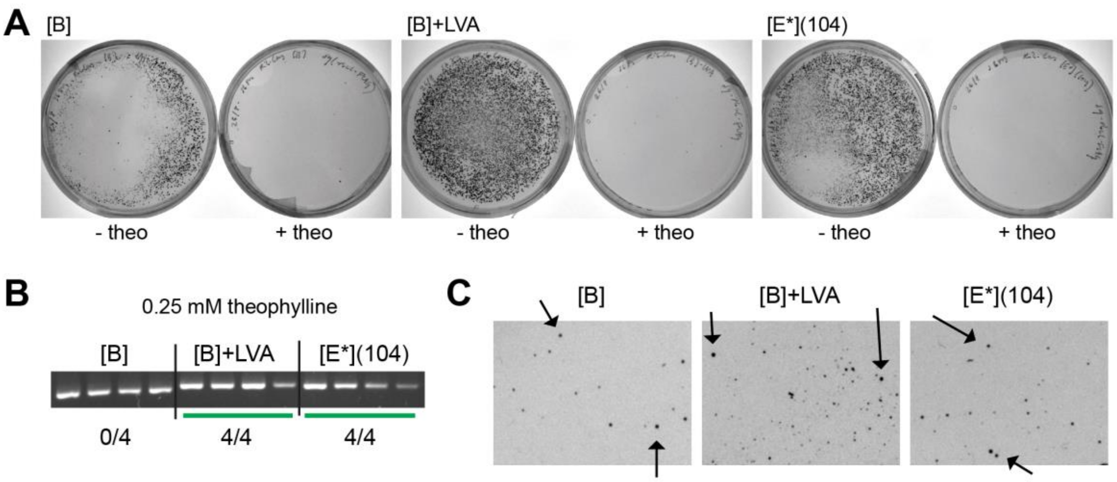

Figure S6. (**A**) Full plates of S6803 wt transformed with *rbcL*-targeting (adding a C-terminal FLAG) pPMQAK1-CRISPR/Cas9 vector variants [B], [B]+LVA, and [E*](104). Selective plates without (-theo) and with 0.25 mM theophylline (+theo) are both shown. (**B**) Screening surviving colonies from the +theo transformation plates seen in (A). A green line below a lane signals a fully edited (+24 bp) mutant. Fractions indicated the number of fully edited colonies out of the total number screened. (**C**) Zoomed-in sections of inducer-plates (0.25 mM), showing the appearance of different sized colonies. The larger ones (examples indicated by arrows) were screened for editing.

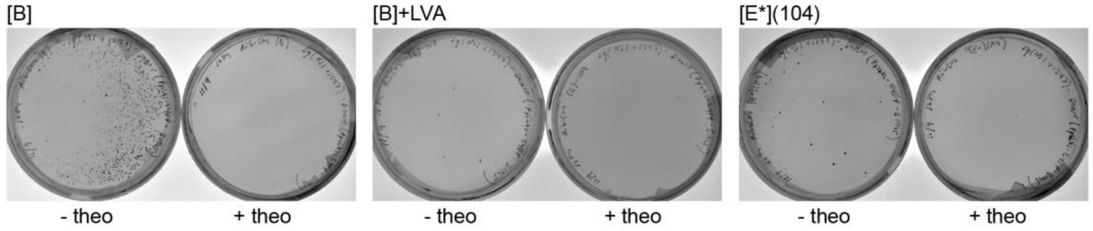

Figure S7. Full plates of S6803 wt transformed with *slr0168*-targeting (+1220 bp, P_{psbA2}-Yfp-B0015 cassette) pPMQAK1-CRISPR/Cas9 vector variants [B], [B]+LVA, and [E*](104). Selective plates without (-theo) and with 0.25 mM theophylline (+theo) are both shown.

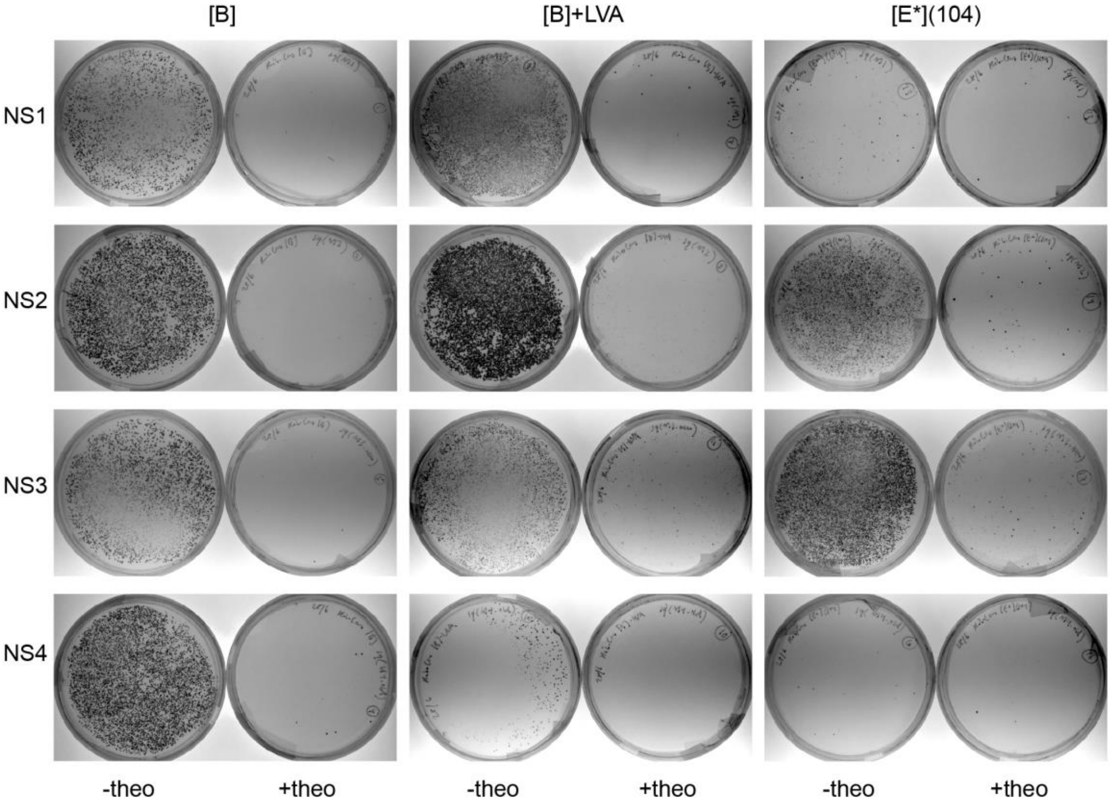

Figure S8. Full plates of S6803 wt transformed with the individually neutral site-targeting pPMQAK1-CRISPR/Cas9 vector variants [B], [B]+LVA, and [E*](104). Selective plates without (-theo) and with 0.25 mM theophylline (+theo) are both shown.

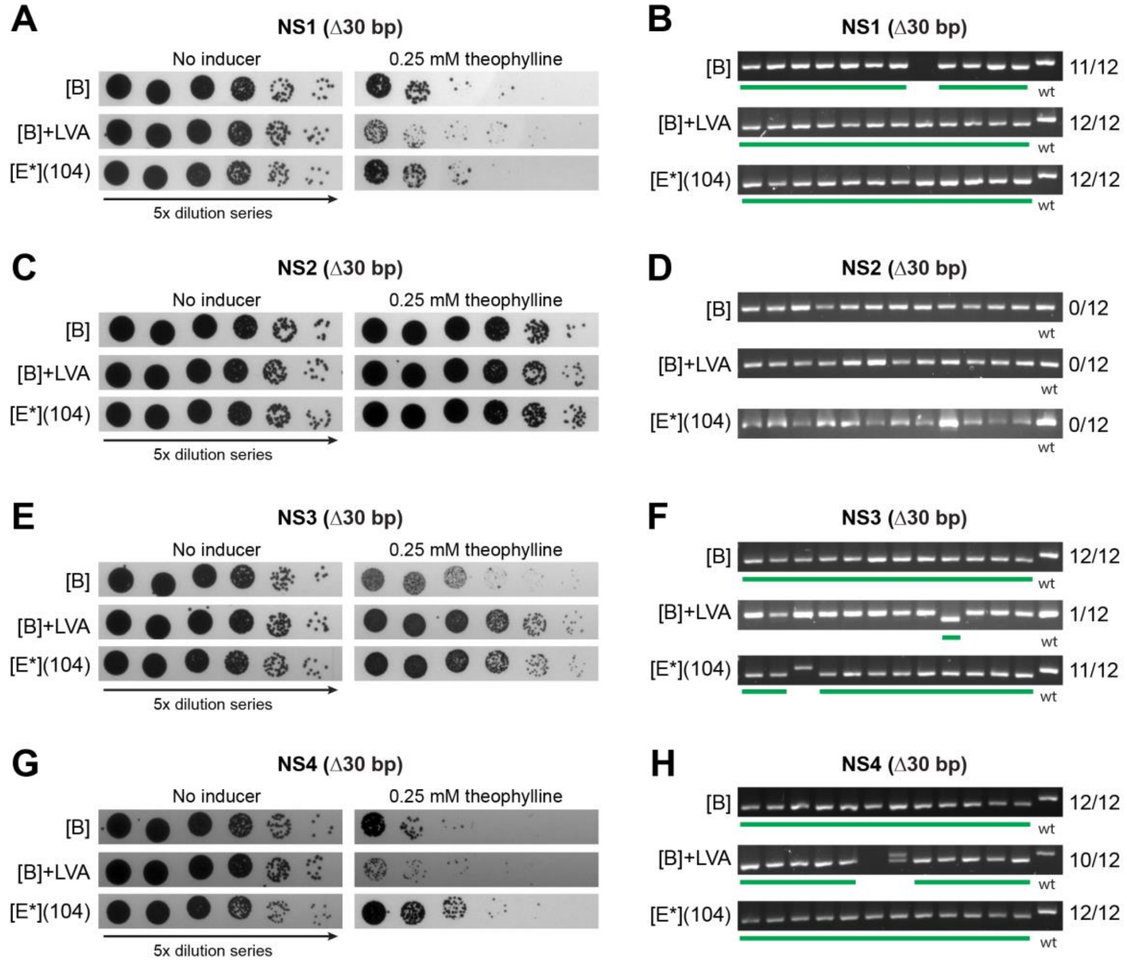

Figure S9. Results from induced editing (Δ30 bp) of NS1-4 as single targets. (**A, C, E, G**) Induction spot assay results for the individual NS1-4 targets, with the indicated pPMQAK1-CRISPR/Cas9 vector variant. 5x dilution series were plated on plates without or with 0.25 mM theophylline. Done for biological duplicates, representative data is shown. (**B, D, F, H**) Editing results for the individual NS1-4 (Δ30 bp) targets, with the indicated CRISPR/Cas9 vector variants. A green line below a lane signals a fully edited (Δ30 bp) mutant. A wt control shows how an unedited colony will appear. Fractions indicated the number of fully edited colonies out of the total number screened.

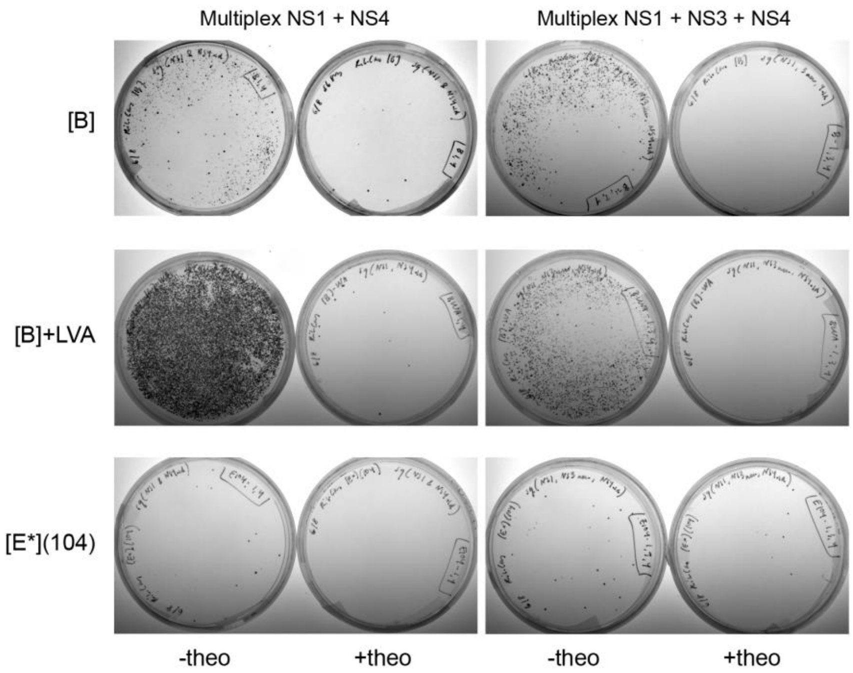

Figure S10. Full plates of S6803 wt transformed with multi-target pPMQAK1-CRISPR/Cas9 vector variants [B], [B]+LVA, and [E*](104). The multiplexed constructs targeted either NS1+NS4, or NS1+NS3+NS4. Selective plates without (-theo) and with 0.25 mM theophylline (+theo) are both shown.

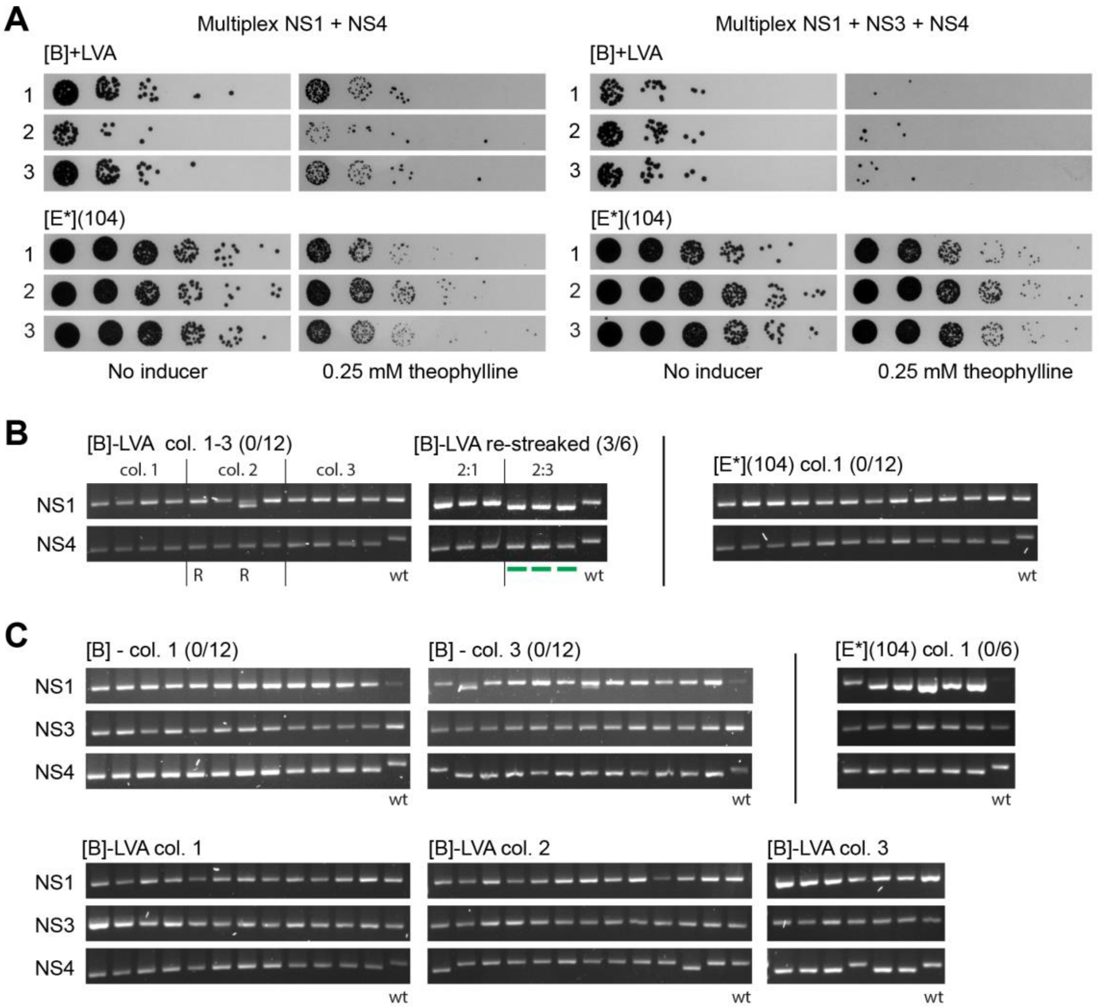

Figure S11. Additional results from induced multi-editing of the combined targets NS1+NS4 and NS1+NS3+NS4 in S6803. (**A**) Induction spot assay results for triplicate transformants of double- and triple-target pPMQAK1-CRISPR/Cas9 vector variants [B]+LVA and [E*](104). 5x dilution series were plated on plates with or without 0.25 mM theophylline. (**B-C**) Editing results for the (B) double-target (NS1+NS4) or (C) triple-target (NS1+NS3+NS4) constructs. The [B], [B]+LVA, and [E*](104) vector variants were screened where indicated. A green line below a lane signals a fully segregated multi-edit in that screened colony. Fractions indicated the number of fully edited colonies out of the total number screened. A wt control shows how an unedited colony will appear. An "R" below a lane indicates a not fully segregated mutant that was re-streaked for a second round of induction.

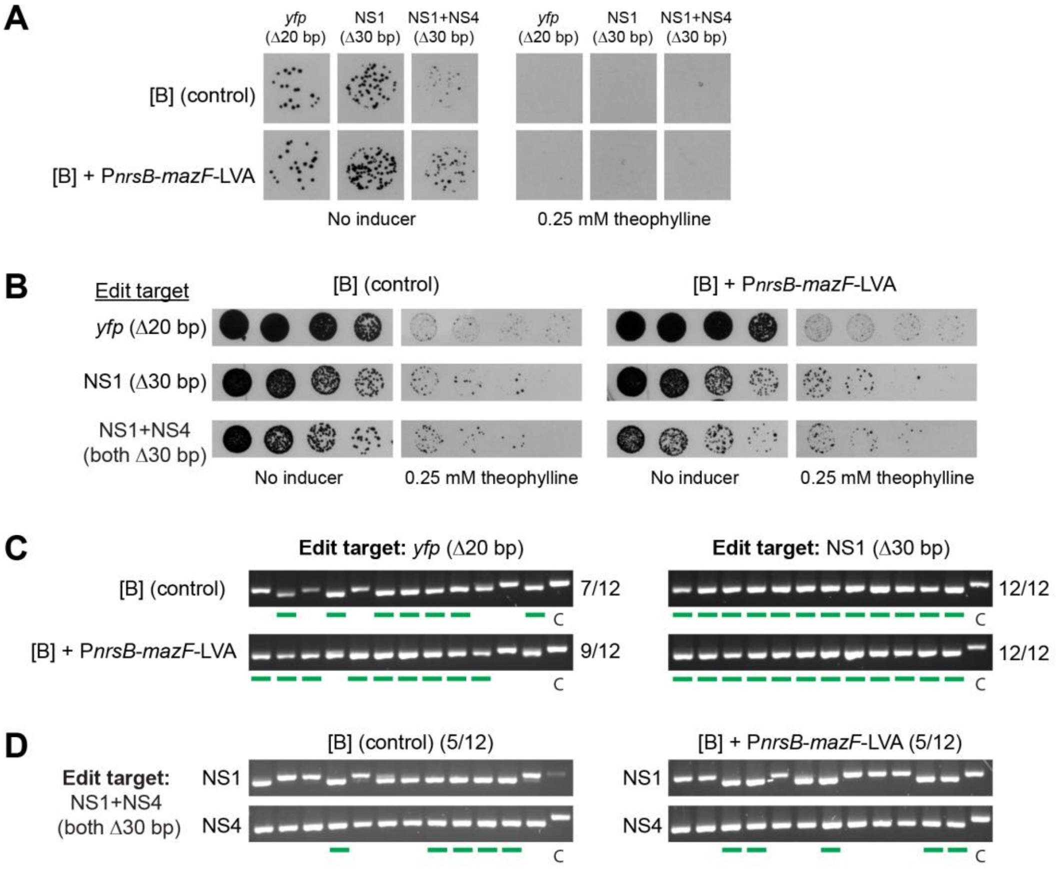

Figure S12. Results from comparing the editing ability of construct [B] when supplemented with P_{nrsB}-*mazF*-LVA or without a curing system (control). Editing of *yfp* (Δ20 bp), NS1 (Δ30 bp), and multiplexed editing of NS1+NS4 (both Δ30 bp) were all tested. (**A**) S6803 transformation results after plating on selective plates, without or with 0.25 mM theophylline. (**B**) Induction spot assay results. 5x dilution series were plated on plates with or without 0.25 mM theophylline. Done for biological triplicates, representative data is shown. (**C**) Editing results for single targets *yfp* (Δ20 bp) and NS1 (Δ30 bp). (**D**) Editing results for multiplexed NS1+NS4 (both Δ30 bp). (**C-D**) A green line below a lane signals a fully edited mutant. Colonies that appear segregated but have not been marked as such, are due to them having detectable wt-bands when the gels are more closely inspected. A control ("C") shows how an unedited colony will appear. Fractions indicated the number of fully edited colonies out of the total number screened.

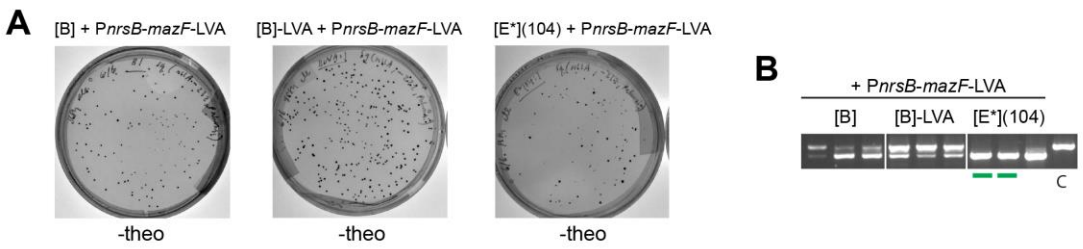

Figure S13. (**A**) Full plates of S6803 wt transformed with *nblA1-2* targeting pPMQAK1-CRISPR/Cas9 vector variants [B], [B]+LVA, and [E*](104), all supplemented with the P_{nrsB}-*mazF*-LVA curing system. Selective plates without theophylline are shown. (**B**) Screening three colonies each from the transformation plates seen in (A), i.e. ones without theophylline inducer, to assess leaky editing. A green line below a lane signals a fully edited (Δ458 bp) mutant. A control ("C") shows how an unedited colony will appear.

Tables

Table S1. Strains and vectors used or constructed in this study. Only CRISPR/Cas9 **base** vectors are shown, the target vectors are not listed. (B0015 is terminator BBa_B0015).

Strain or Vectors	Relevant characteristics	Source
**Strains**		
Escherichia coli XL1-Blue	Cloning host	Stratagene
Synechocystis sp. PCC 6803		
wild type	Non-motile, GT-S derivative	M. Fulda
Δ*slr1181*::P_{psbA2}-Yfp-B0015-Sp^r	Yfp expressed from P*psbA2* in site *slr1181*, Sp^r	In-lab, D. Kaczmarzyk
**Vectors**		
pPMQAK1-T	Replicative vector (RSF1010). Amp^r, Km^r	Vasudevan et al.[1]
pPMQAK1-T (no BsaI)	Replicative vector (RSF1010), BsaI site removed. Amp^r, Km^r	This study
pPMQAK1-P_{conII}-[B]-Gfp-B0015	Gfp-reporter vector, Amp^r, Km^r	This study
pPMQAK1-P_{conII}-[C]-Gfp-B0015	Gfp-reporter vector, Amp^r, Km^r	This study
pPMQAK1-P_{conII}-[E*]-Gfp-B0015	Gfp-reporter vector, Amp^r, Km^r	This study
pPMQAK1-P_{trc}-[B]-Gfp-B0015	Gfp-reporter vector, Amp^r, Km^r	This study
pPMQAK1-P_{trc}-[C]-Gfp-B0015	Gfp-reporter vector, Amp^r, Km^r	This study
pPMQAK1-P_{trc}-[E*]-Gfp-B0015	Gfp-reporter vector, Amp^r, Km^r	This study
pPMQAK1-P_{trc}-[B]-Cas9-B0015-*lacZ*	CRISPR/Cas9 base vector [B], Amp^r, Km^r	This study
pPMQAK1-P_{trc}-[C]-Cas9-B0015-*lacZ*	CRISPR/Cas9 base vector [C], Amp^r, Km^r	This study
pPMQAK1-P_{trc}-[E*]-Cas9-B0015-*lacZ*	CRISPR/Cas9 base vector [E*], Amp^r, Km^r	This study
pPMQAK1-P_{trc}-[B]-Cas9+LVA-B0015-*lacZ*	CRISPR/Cas9 base vector [B]+LVA, Amp^r, Km^r	This study
pPMQAK1-P_{trc}-[C]-Cas9+LVA-B0015-*lacZ*	CRISPR/Cas9 base vector [C]+LVA, Amp^r, Km^r	This study
pPMQAK1-P_{trc}-[E*]-Cas9+LVA-B0015-*lacZ*	CRISPR/Cas9 base vector [E*]+LVA, Amp^r, Km^r	This study
pPMQAK1-P_{trc}(-10:TATTGT)-[B]-Cas9-B0015-*lacZ*	CRISPR/Cas9 base vector [B](104), Amp^r, Km^r	This study
pPMQAK1-P_{trc}(-10:TATTGT)-[C]-Cas9-B0015-*lacZ*	CRISPR/Cas9 base vector [C](104), Amp^r, Km^r	This study
pPMQAK1-P_{trc}(-10:TATTGT)-[E*]-Cas9-B0015-*lacZ*	CRISPR/Cas9 base vector [E*](104), Amp^r, Km^r	This study
pPMQAK1-P_{trc}(-10:GACTAT)-[B]-Cas9-B0015-*lacZ*	CRISPR/Cas9 base vector [B](116), Amp^r, Km^r	This study
pPMQAK1-P_{trc}(-10:GACTAT)-[C]-Cas9-B0015-*lacZ*	CRISPR/Cas9 base vector [C](116), Amp^r, Km^r	This study
pPMQAK1-P_{trc}(-10:GACTAT)-[E*]-Cas9-B0015-*lacZ*	CRISPR/Cas9 base vector [E*](116), Amp^r, Km^r	This study
pPMQAK1-P_{trc}(-10:GATTGT)-[B]-Cas9-B0015-*lacZ*	CRISPR/Cas9 base vector [B](117), Amp^r, Km^r	This study
pPMQAK1-P_{trc}(-10:GATTGT)-[C]-Cas9-B0015-*lacZ*	CRISPR/Cas9 base vector [C](117), Amp^r, Km^r	This study

pPMQAK1-P_{trc}(-10:GATTGT)-[E*]-Cas9-B0015-*lacZ*	CRISPR/Cas9 base vector [E*](117), Amp^r, Km^r	This study
pPMQAK1-Cas9-B0015-*lacZ*	Control vector, Cas9 lacks promoter and ATG, Amp^r, Km^r	This study
pPMQAK1-P_{trc}-[B]-Cas9-B0015-*lacZ*-P_{nrsB}-*mazF*-LVA	CRISPR/Cas9 base vector [B], inducible *mazF*-LVA, Amp^r, Km^r	This study
pPMQAK1-P_{trc}-[B]-Cas9-B0015-*lacZ*-P_{nrsB}-*mazF*-AAV	CRISPR/Cas9 base vector [B], inducible *mazF*-AAV, Amp^r, Km^r	This study
pPMQAK1-P_{trc}-[B]-Cas9-B0015-*lacZ*-P_{nrsD}-*mazF*-LVA	CRISPR/Cas9 base vector [B], inducible *mazF*-LVA, Amp^r, Km^r	This study
pPMQAK1-P_{trc}-[B]-Cas9-B0015-*lacZ*-P_{nrsD}-*mazF*-AAV	CRISPR/Cas9 base vector [B], inducible *mazF*-AAV, Amp^r, Km^r	This study
pPMQAK1-P_{trc}-[B]-Cas9+LVA-B0015-*lacZ*-P_{nrsB}-*mazF*-LVA	CRISPR/Cas9 base vector [B]+LVA, inducible *mazF*-LVA, Amp^r, Km^r	This study
pPMQAK1-P_{trc}-[E*](104)-Cas9-B0015-*lacZ*-P_{nrsB}-*mazF*-LVA	CRISPR/Cas9 base vector [E*](104), inducible *mazF*-LVA, Amp^r, Km^r	This study
pMD19-(EcoRI, XbaI)-Sp^r	Vector with cloning sites, for building sgRNA-array template vectors (below), Sp^r	In-lab, L. Yao
pMD19-(EcoRI, XbaI)-Cm^r	Vector with cloning sites, for building sgRNA-array template vectors (below), Cm^r	In-lab, L. Yao
pMD19-BsaI-P_{BBa_J23117}-sgRNA-BsaI-Sp^r	Template vector – sgRNA single and multiplex-array (as Li et al.),[2] flanking BsaI-sites added, Sp^r	This study
pMD19-Cas9_handle-*S.pyogenes*_terminator-P_{BBa_J23117}-Cm^r	Template vector – sgRNA multiplex-array (as Li et al.),[2] Cm^r	This study

Table S2. Primers used in this study.

Name	Sequence (5' to 3')	Description
Construction of Gfp-reporters [a]		
IVCE572	**CATGTGGAAGACAGTGCC**ACCGGTTTCGAATTGACA	fwd - PconII (BpiI)
IVCE1120	**TCACTGGAAGACAGTGCC**AAATATTCTGAAATGAGCTGTTGA	fwd - Ptrc (BpiI)
IVCE637	**CATGTGGAAGACAGACG**CATCTTGTTGATACCCCCT	rev - riboswitch [B] (BpiI)
IVCE576	**TCTCCTGAAGACGTACG**CATCTTGTTGTCCCTTGGT	rev - riboswitch C (BpiI)
IVCE578	**TCACGTGAAGACAGACG**CATCTTGTTGCCTCCTTAGC	rev - riboswitch E* (BpiI)
IVCE574	**CATGTGGAAGACCAG**CGTAAAGGAGAAGAACTTTTCA	fwd - Gfp-B0015 (BpiI)
IVCE575	**CATGTGGAAGACCATCCC**TATAAACGCAGAAAGGCCC	rev - Gfp-B0015 (BpiI)
Domestication of BsaI site in pPMQAK1-T [b]		
IVCE721	CGCGAGATCCACGCTCACCGGCTCC	fwd - mutating BsaI-site in pPMQAK1 (in Amp^r)
IVCE722	AGCGTGGATCTCGCGGTATCATTGCAGCAC	rev - mutating BsaI-site in pPMQAK1 (in Amp^r)

Domestication of BpiI sites in *cas9* [a, b]

IVCE711	**ACGCAAGAAGACAGA**GGTTTCTTAGACGTCAGGT	fwd - pMD19 (BpiI)
IVCE712	**ACGCAAGAAGACAGGT**TCCACACAACATACGAGC	rev - pMD19 (BpiI)
IVCE713	**ACGCAAGAAGACCAGA**ACCGGTTTCGAATTGACA	fwd - PconII-Cas9 Piece 1 (BpiI)
IVCE714	**ACGCAAGAAGACCACTT**ATCTTCTTCCACCAAAAAAGAC	rev - PconII-Cas9 Piece 1 (BpiI)
IVCE715	**ACGCAAGAAGACCAT**AAGAAGCATGAACGTCATC	fwd - Cas9 Piece 2 (BpiI)
IVCE716	**ACGCAAGAAGACCATT**GCCTTCTCAAAATAGCATG	rev - Cas9 Piece 2 (BpiI)
IVCE717	**ACGCAAGAAGACCTGCA**AGAGGACTTTTATCCATTTTTAAAAG	fwd - Cas9 Piece 3 (BpiI)
IVCE718	**ACGCAAGAAGACCTT**ATCTTCTTTAAATGTCAAACTATCATC	rev - Cas9 Piece 3 (BpiI)
IVCE719	**ACGCAAGAAGACTGGAT**ATTCAAAAAGCACAAGTGTCT	fwd - Cas9 Piece 4 (BpiI)
IVCE720	**ACGCAAGAAGACTGACC**TTAGTCACCTCCTAGCTGA	rev - Cas9 Piece 4 (BpiI)

Construction of pPMQAK1-CRISPR/Cas9 base vectors [a, c]

IVCE1120	**TCACTGGAAGACAGTGCC**AAATATTCTGAAATGAGCTGTTGA	fwd - Ptrc (BpiI)
IVCE1121	**TCACTGGAAGACAGC**CATCTTGTTGATACCCCCT	rev - Ptrc + constant region + [B] + ATG (BpiI)
IVCE1128	**GGATTGGAAGACCTC**CATCTTGTTGTCCCTTGGT	rev - Ptrc + constant region + [C] + ATG (BpiI)
IVCE1129	**GCCATAGAAGACCTC**CATCTTGTTGCCTCCTTAGC	rev - Ptrc + constant region + [E*] + ATG (BpiI)
IVCE1122	**TCACTGGAAGACAGATG**GATAAGAAATACTCAATAGGCTTAGA	fwd - Cas9 (no start) (BpiI)
IVCE1123	**TCACTGGAAGACAGGTTA**TTAGTCACCTCCTAGCTGA	rev - Cas9 (extra stop) (BpiI)
IVCE1124	**TCACTGGAAGACGTTAA**CCAGGCATCAAATAAAACGA	fwd - B0015 (BpiI)
IVCE1125	**TCACTGGAAGACGTACCTCTAGTA**TATAAACGCAGAAAGGCCCAC	rev - B0015 + generic extra bp (BpiI)
IVCE1126	**TCACTGGAAGACAGAG**GTTTGGAGACCACGTGTTCACAGCTTGTCTGTAAGCG	fwd - lacZ + (BsaI site for future use) (BpiI)
IVCE1127	**TCACTGGAAGACAGTCCC**TGTGCCACGAGACCACGTGTGCAGCTGGCACGACAGGTTT	rev - lacZ + (BsaI site for future use) (BpiI)
IVCE1132	**GCGTTAGAAGACGATGCC**GATAAGAAATACTCAATAGGCTTAGA	fwd - Cas9 (no start), for promoterless & ATG-less control construct (BpiI)

Mutagenesis of pPMQAK1-CRISPR/Cas9 base vectors to obtain extra variants [b]

IVCE1150	CGACGAAAACTACGCTTTAGTAGCTTAATAACCAGGCATCAAATAAAACGAA	fwd - add LVA-tag to Cas9
IVCE1084	AGCGTAGTTTTCGTCGTTTGCAGCGTCACCTCCTAGCTGACTCA	rev - add LVA-tag to Cas9
IVCE1151	GCTCGTATTGTGTGTGGAATTGTGAGCGG	fwd - mutate Ptrc -10-box (to one in BBa_J23104: TATAAT->TATTGT)

IVCE1152	CCACACACAATACGAGCCGGATGATTAATTGTC	rev - mutate Ptrc -10-box (to one in BBa_J23104: TATAAT->TATTGT)
IVCE1153	GGCTCGGACTATGTGTGGAATTGTGAGCGG	fwd - mutate Ptrc -10-box (to one in BBa_J23116: TATAAT->GACTAT)
IVCE1154	CACACATAGTCCGAGCCGGATGATTAATTGTCA	rev - mutate Ptrc -10-box (to one in BBa_J23116: TATAAT->GACTAT)
IVCE1155	GGCTCGGATTGTGTGTGGAATTGTGAGCGG	fwd - mutate Ptrc -10-box (to one in BBa_J23117: TATAAT->GATTGT)
IVCE1156	CACACACAATCCGAGCCGGATGATTAATTGTCA	rev - mutate Ptrc -10-box (to one in BBa_J23117: TATAAT->GATTGT)
Construction of donor DNA pieces [a]		
IVCE1144	**AACGTCGGTCTCGTTCG**GCGATGTAAACGGCCACAAA	fwd - H1 (yfp Δ20 bp) (BsaI)
IVCE1145	**TATGGCGGATCTTG**CTTCTGCTTATCGGCCATAA	rev - H1 (yfp Δ20 bp) (overhang overlaps with H2)
IVCE1146	**CCGATAAGCAGAAG**CAAGATCCGCCATAACATCG	fwd - H2 (yfp Δ20 bp) (overhang overlaps with H1)
IVCE1147	**ACACGTGGTCTCGGCCA**AGTAGAGAGCGTTCACCGAC	rev - H2 (yfp Δ20 bp) (BsaI)
IVCE1192	**AACGTCGGTCTCGTTCG**AGGGTGTTGGCCAAGGTA	fwd - H1 (yfp Δ2240 bp) (BsaI)
IVCE1193	**GCATCACCGGAAGCTAAA**TGGTTTCTCAGATTGCAGTTG	rev - H1 (yfp Δ2240 bp) (overhang overlaps with H2)
IVCE1194	**CTGCAATCTGAGAAACCA**TTTAGCTTCCGGTGATGCCC	fwd - H2 (yfp Δ2240 bp) (overhang overlaps with H1)
IVCE1195	**ACACGTGGTCTCGGCCA**GATTTTGTTCAGCTAGTAACTG	rev - H2 (yfp Δ2240 bp) (BsaI)
IVCE1326	**AACGTCGGTCTCGTTCG**CCCGTAGCTTCCGGTGGTAT	fwd - H1 (rbcL+FLAG) (BsaI)
IVCE646	**CATTGGCTTACTTATCGTCATCATCCTTGTAGTC**GAGGGTATCCATGGCCTCGA	rev - H1 (rbcL+FLAG) (overhang overlaps with H2)
IVCE647	**TCGACTACAAGGATGATGACGATAAGTAAGCCAA**TGTTTGGATTGTCGGAGTTGT	fwd - H2 (rbcL+FLAG) (overhang overlaps with H1)
IVCE1327	**ACACGTGGTCTCGGCCA**TTCTTTATTTTCATCCAGGAGTTCC	rev - H2 (rbcL+FLAG) (BsaI)
IVCE1212	**AACGTCGGTCTCGTTCG**GGGACGGACAGTTATCCTAA	fwd - H1 (NS1: Yfp-insertion and Δ30 bp) (BsaI)
IVCE1328	**TACCGAGGTCTCGTT**AGGAGACTTTGGTGGGCT	rev - H1 (NS1: Yfp-insertion) (BsaI)
IVCE1329	**TACCGAGGTCTCCCT**AACTGACTGACCACTGAC	fwd - PpsbA2-Yfp-B0015 (for insertion into NS1 (BsaI)
IVCE1330	**TACCGAGGTCTCCGAT**TATAAACGCAGAAAGGCCC	rev - PpsbA2-Yfp-B0015 (for insertion into NS1 (BsaI)
IVCE1331	**TACCGAGGTCTCCA**ATCCCTTCAGTGGTACTCC	fwd - H2 (NS1: Yfp-insertion) (BsaI)
IVCE1215	**ACACGTGGTCTCGGCCA**ACCTACCTGTCCTGGGTTGA	rev - H2 (NS1: Yfp-insertion and Δ30 bp) (BsaI)
IVCE1213	**ACCACTGAAGGGAT**AGGAGACTTTGGTGGGCTG	rev - H1 (NS1 Δ30 bp) (overhang overlaps with H2)
IVCE1214	**CACCAAAGTCTCCT**ATCCCTTCAGTGGTACT	fwd - H2 (NS1 Δ30 bp) (overhang

	CC	overlaps with H1)
IVCE1216	**AACGTCGGTCTCGTTCG**ATTGATGGCATTCGGGAGCC	fwd - H1 (NS2 Δ30 bp) (BsaI)
IVCE1217	**TATGTTCCGCTTGC**ATGCCTAGGGGTAAACCATC	rev - H1 (NS2 Δ30 bp) (overhang overlaps with H2)
IVCE1218	**TTACCCCTAGGCAT**GCAAGCGGAACATAACGTAT	fwd - H2 (NS2 Δ30 bp) (overhang overlaps with H1)
IVCE1219	**ACACGTGGTCTCGGCCA**TGAAGTTAAAACCGTTGAGG	rev - H2 (NS2 Δ30 bp) (BsaI)
IVCE1266	**AACGTCGGTCTCGTTCG**AAAGTTGGGCACAACCATTT	fwd - H1 (NS3 Δ30 bp) (BsaI)
IVCE1267	**GTAAACTCCGCAGGG**CTTTATTTTGTTCCCGGTAC	rev - H1 (NS3 Δ30 bp) (overhang overlaps with H2)
IVCE1268	**CCGGGAACAAAATAAAG**CCCTGCGGAGTTTACATAATC	fwd - H2 (NS3 Δ30 bp) (overhang overlaps with H1)
IVCE1269	**ACACGTGGTCTCGGCCA**CAGCTATAGTTACGATCTTG	rev - H2 (NS3 Δ30 bp) (BsaI)
IVCE1224	**AACGTCGGTCTCGTTCG**CTATCCTATGCATGTCATGT	fwd - H1 (NS4 Δ30 bp) (BsaI)
IVCE1225	**CGTACTCGCTTAAA**TCCTTGGTCTGTTCAACTAA	rev - H1 (NS4 Δ30 bp) (overhang overlaps with H2)
IVCE1226	**GAACAGACCAAGGA**TTTAAGCGAGTACGCCCGAA	fwd - H2 (NS4 Δ30 bp) (overhang overlaps with H1)
IVCE1227	**ACACGTGGTCTCGGCCA**CATAGATATTTGCCCAGTTA	rev - H2 (NS4 Δ30 bp) (BsaI)
IVCE1315	**GGTTACGGTCTCCAG**ACCTACCTGTCCTGGGTT	rev - H2 NS1 (Δ30 bp), multi: compatible with donor NS4 (Δ30 bp) (BsaI)
IVCE1316	**GGTTACGGTCTCCGT**CTATCCTATGCATGTCATGTG	fwd - H1 NS4 (Δ30 bp), multi: compatible with donor NS1 (Δ30 bp) (BsaI)
IVCE1313	**GTTTCGGGTCTCGTT**ACCTACCTGTCCTGGGTT	rev - H2 (NS1 Δ30 bp), multi: compatible with donor NS3 (Δ30 bp) (BsaI)
IVCE1314	**GTTTCGGGTCTCCGT**AAAGTTGGGCACAACCAT	fwd - H1 (NS3 Δ30 bp), multi: compatible with donor NS1 (Δ30 bp) (BsaI)
IVCE1277	**GCATCTGGTCTCGAG**CAGCTATAGTTACGATCTTG	rev – H2 (NS3 Δ30 bp), multi: compatible with donor NS4 (Δ30 bp) (BsaI)
IVCE1241	**GCATCTGGTCTCGTG**CTATCCTATGCATGTCATGTG	fwd – H1 (NS4 Δ30 bp), multi: compatible with donor NS3 (Δ30 bp) (BsaI)
IVCE1435	**AACGTCGGTCTCGTTCG**CACCCAAGGAAATGCATTACTT	fwd - H1 (nblA KO) (BsaI)
IVCE1436	**TTGGAGGGCAACAGCT**CAACTCTAGGTTTAGGCATCAAGC	rev - H1 (nblA KO) (overhang overlaps with H2)
IVCE1437	**AACCTAGAGTTG**AGCTGTTGCCCTCCAAGG	fwd - H2 (nblA KO) (overhang overlaps with H1)
IVCE1438	**ACACGTGGTCTCGGCCA**ATAGCCCTGAGCACCAGAAG	rev - H2 (nblA KO) (BsaI)
Construction of sgRNA pieces [a, d]		
IVCE992	**ACACGTGGTCTCGGTTT**TTGACAGCTAGCTCAGTCCT	fwd – BBa_J23117-sgRNA, to amplify whole sgRNA (BsaI)
IVCE993	**AACGTCGGTCTCGCGAA**AAAAAAAGCACC	rev – sgRNA scaffold, to amplify

	GACTCGGTGC	whole sgRNA (BsaI)
IVCE1142	CTATGGCCGATAAGCAGAAGAATGTTTTAGAGCTAGAAATAGCAAGT	fwd - Cas9-handle (overhang: yfp-spacer)
IVCE1143	AACATTCTTCTGCTTATCGGCCATAGCTAGCACAATCCCTAGG	rev – BBa_J23117 (overhang: yfp-spacer)
IVCE645	CTAGGCCATGGATACCCTCTAAACGTTTTAGAGCTAGAAATAGCAAGT	fwd - Cas9-handle (overhang: rbcL-spacer)
IVCE644	AACGTTTAGAGGGTATCCATGGCCTAGCTAGCACAATCCCTAGGACT	rev – BBa_J23117 (overhang: rbcL-spacer)
IVCE1204	CTAGCCCACCAAAGTCTCCTATGGTTTTAGAGCTAGAAATAGCAAGT	fwd - Cas9-handle (overhang: NS1-spacer)
IVCE1205	AACCATAGGAGACTTTGGTGGGCTAGCTAGCACAATCCCTAGG	rev – BBa_J23117 (overhang: NS1-spacer)
IVCE1206	CTATGGTTTACCCCTAGGCATCAGGTTTTAGAGCTAGAAATAGCAAGT	fwd - Cas9-handle (overhang: NS2-spacer)
IVCE1207	AACCTGATGCCTAGGGGTAAACCATAGCTAGCACAATCCCTAGG	rev – BBa_J23117 (overhang: NS2-spacer)
IVCE1262	CTACCGGGAACAAAATAAAGTGCGTTTTAGAGCTAGAAATAGCAAGT	fwd - Cas9-handle (overhang: NS3-spacer)
IVCE1263	AACGCACTTTATTTTGTTCCCGGTAGCTAGCACAATCCCTAGG	rev – BBa_J23117 (overhang: NS3-spacer)
IVCE1210	CTAGTTGAACAGACCAAGGAACAGTTTTAGAGCTAGAAATAGCAAGT	fwd - Cas9-handle (overhang: NS4-spacer)
IVCE1211	AACTGTTCCTTGGTCTGTTCAACTAGCTAGCACAATCCCTAGG	rev – BBa_J23117 (overhang: NS4-spacer)
IVCE1432	CTACGGTCAAAAATTGTAACTAAGTTTTAGAGCTAGAAATAGCAAGT	fwd - Cas9-handle (overhang: nblA spacer)
IVCE1434	AACTTAGTTACAATTTTTGACCGTAGCTAGCACAATCCCTAGG	rev – BBa_J23117 (overhang: nblA spacer)

Constructing sgRNA-array template vectors as described in Li et al.[2] [e]

IVCE851	**CTAGAATTCGCGGCCGCTTCTAGA**ACACGTGGTCTCGGTTTTTGACAGCTAGCTCAGTC	fwd – BBa_J23117-sgRNA, adds BsaI (EcoRI+NotI+XbaI)
IVCE852	**CTGCAGCGGCCGCTACTAGT**AACGTCGGTCTCGCGAAAAAAAAAGCACCGACTCG	rev – sgRNA scaffold, adds BsaI (PstI+NotI+BcuI)
IVCE853	**CTAGAATTCGCGGCCGCTTCTAGA**GTTTTAGAGCTAGAAATAGCAAG	fwd - Cas9-handle (EcoRI+NotI+XbaI)
IVCE854	**CTGCAGCGGCCGCTACTAGT**TAGCTAGCACAATCCCTAGGACTGAGCTAGCTGTCAAAAAAAAAGCACCGACTCGGTGC	rev – sgRNA scaffold, adds BBa_J23117 overhang (PstI+NotI+BcuI)

For sgRNA-array construction (multiplex targeting), as described in Li et al.[2] [a, d]

IVCE1228	**GGACTAGAAGACGAT**AGTTGAACAGACCAAGGAACAGTTTTAGAGCTAGAAATAGCAAGT	fwd - Cas9-handle (in pMD19 template vector), adds NS4-spacer (BpiI)
IVCE1229	**GGACTAGAAGACGAGGC**TAGCTAGCACAATCCCTAGG	rev – BBa_J23117 (in pMD19 template vector), overhang compatible with NS1-spacer (BpiI)
IVCE1230	**GGACTAGAAGACTG**AGCCCACCAAAGTCTCCTATGGTTTTAGAGCTAGAAATAGCAAGT	fwd - Cas9-handle, adds NS1-spacer (BpiI)
IVCE1235	**GGACTAGAAGACGAAC**TAGCTAGCACAATCCCTAGG	rev – BBa_J23117, overhang compatible with NS4-spacer (BpiI)
IVCE1273	**TAACCCGAAGACAGGG**TAGCTAGCACAATCCCTAGG	rev – BBa_J23117, overhang compatible with NS3-spacer

		(BpiI)
IVCE1274	**TAACCCGAAGACTCT**ACCGGGAACAAAATAAAGTGCGTTTTAGAGCTAGAAATAGCAAGT	fwd - Cas9-handle, adds NS3-spacer (BpiI)
Construction of *mazF*-based curing system pieces [a]		
IVCE1421	**TGAGGTGAAGACGTAC**TGTGCCACGAGACCACGT	rev – *lacZ* (compatible with downstream P_{nrsB}) (BpiI)
IVCE1422	**GTGTGAGAAGACAGTA**TGTGCCACGAGACCACGT	rev – *lacZ* (compatible with downstream P_{nrsD}) (BpiI)
IVCE1423	**TGAGGTGAAGACGACA**GTCTGATCTTAGCGGGGG	fwd - P_{nrsB} (BpiI)
IVCE1424	**TGAGGTGAAGACGAAT**ACCACCTCAAATTGGGAA	rev - P_{nrsB} (BpiI)
IVCE1425	**GTGTGAGAAGACAGCA**TATTCGATTCAGTACCAAGTACT	fwd - P_{nrsD} (BpiI)
IVCE1426	**GTGTGAGAAGACAGAT**TGTGGGGGTTTGGGGTAG	rev - P_{nrsD} (BpiI)
IVCE1427	**TGAGGTGAAGACGAGT**ATGGTAAGCCGATACGTAC	fwd – *mazF* (compatible with upstream P_{nrsB}) (BpiI)
IVCE1428	**GTGTGAGAAGACGTCA**ATGGTAAGCCGATACGTAC	fwd – *mazF* (compatible with upstream P_{nrsD}) (BpiI)
IVCE1429	**TGAGGTGAAGACGATCCC**CTAAGCTACTAAAGCGTAGTTTTCGTCGTTTGCAGCCCCAATCAGTACGTTAATTTTGGC	rev – *mazF* + LVA-tag (in red) (BpiI)
IVCE1430	**GTGTGAGAAGACGTTCCC**CTATACGGCAGCAGCGTAGTTTTCGTCGTTTGCAGCCCCAATCAGTACGTTAATTTTGGC	rev – *mazF* + AAV-tag (in red) (BpiI)
Colony-PCR screening primers		
IVCE799	ATGGTATCCAAAGGCGAGGAG	fwd - yfp Δ20 bp
IVCE1170	TTGCACACTTCCGTCTTCGA	rev - yfp Δ20 bp
IVCE141	TCGATGGAGGGTCAGACCAT	fwd - yfp Δ2240 bp
IVCE142	TCAGCAAAACTGCCGCAATC	rev - yfp Δ2240 bp
IVCE663	GCCGTAGTTGACCGTCAGAA	fwd - rbcL-FLAG
IVCE662	GCAGACTCAACCCCGAAGAA	rev - rbcL-FLAG
IVCE1278	ATTTACACCAGCGCCGGTTT	fwd - NS1 (Yfp and Δ30 bp)
IVCE1279	ACCGCTAAACCCACCTCTTG	rev - NS1 (Yfp and Δ30 bp)
IVCE1280	GCTGGTTTGGGGTGTTGATG	fwd - NS2 Δ30 bp
IVCE1281	ACCCCAGCTACCCCTAACAT	rev - NS2 Δ30 bp
IVCE1323	TGGTGGCTAAGTTGTACCGG	fwd - NS3 Δ30 bp
IVCE1324	AGTAACTTACGACGGGTGGG	rev - NS3 Δ30 bp
IVCE1284	TACCAAGAACTACTGCGGCG	fwd - NS4 Δ30 bp
IVCE1285	CTGGGTCGATCTTCCTTCCA	rev - NS4 Δ30 bp
IVCE174	GGAGAAAACTCACCGAGGCA	fwd - Km^r screening (curing)
IVCE175	CTCAGGCGCAATCACGAATG	rev - Km^r screening (curing)
IVCE1475	CTTGTTAATGACCGCCGGGA	fwd - nblA Δ458 bp
IVCE1476	GGGATGTATAACCCTGGGCG	rev - nblA Δ458 bp

[a] Golden Gate cloning compatible overhangs are in bold. See description column for specification of the type II restriction enzyme in question.
[b] Mutated nucleotide indicated in red.
[c] Underlined sequences shows the BsaI-containing extra region added to the CRISPR/Cas9 base vectors, for use when constructing the target vector.
[d] Underlined sequences are the added sgRNA spacers.
[e] Overhangs with BioBrick cloning sites are indicated in bold, restriction sites are specified in the description column. Underlined indicates the added BsaI-containing regions added to flank the

finished sgRNA-array. In IVCE854 the BBa_J23117 promoter added after the sgRNA scaffold (Cas9-handle and S. pyogenes terminator) is also underlined.

Table S3. Details for all tested sgRNA-spacers.

Target	Spacer sequence (5'-3')	PAM	On-target score [a]
yfp	ATGGCCGATAAGCAGAAGAAT	GGG	45.8
rbcL	AGGCCATGGATACCCTCTAAAC	CGG	44.2
NS1 (*slr0168*)	AGCCCACCAAAGTCTCCTATG	TGG	62.5
NS2 (*slr1181*)	ATGGTTTACCCCTAGGCATCAG	TGG	62.0
NS3 (*slr2030-2031*)	ACCGGGAACAAAATAAAGTGC	AGG	57.5
NS4 (*slr0397*)	AGTTGAACAGACCAAGGAACA	GGG	73.0
nblA [b]	ACGGTCAAAAATTGTAACTAA	TGG	46.7

[a] The on-target score as determined by Benchling.[3] This score is determined by using an algorithm based on mammalian cell data, it also doesn't consider the position of the spacer in the target gene.[4] However, note that this scoring might not be directly applicable for a prokaryotic host.[5]

[b] An "A" was added at the TSS-position of this sgRNA to accommodate for the potential need for this by PBBa_J23117; this base is thus not present in the target region.

Table S4. Off-target analysis of sgRNAs used in this study, against the S6803 genome, using the CasOT software.[6] The PAM was specified to NGG only.

	Target						
Type [a]	*yfp*	*rbcL*	NS1	NS2	NS3	NS4	*nblA*
A06							
A13							
A14						1	
A15						1	
A16	1			1	1	1	1
A17					1	1	
A18	4	1					
A19	1						
A110							
A23	1	1					
A24	4	1	2		2	2	6
A25	8		2	3	6	8	8
A26	10	1	4	3	10	9	5
A27	22	4	3	9	14	8	5
A28	19	6	6	2	11	5	3
A29	7	3	1	2	7		

[a] Indicates the type of mismatch. The first number (0-2) specifies the number of mismatches in the seed region (12 nt proximal to PAM) of the binding sequence. The following number indicates the mismatches in the remaining non-seed region.

Table S5. Raw data for the calculated CFUs for selected CRISPR/Cas9 editing experiments. The CFUs were calculated from spot-plates (unless otherwise noted), from spots with dispersed enough colonies. The dilution featured in those spots were then used to back-calculate to get the “un-diluted” CFUs, which was used to calculate the percentage of total plated and induced CFUs that survived (i.e. retained a healthy phenotype) and became edited.

	No theophylline [a]									0.25 mM theophylline [b]								
**Target**	**[B]**			**[B]+LVA**			**[E*](104)**			**[B]**			**[B]+LVA**			**[E*](104)**		
yfp Δ20 [c]	20,<br>16	16,<br>16	16,<br>24	26,<br>17	19,<br>16	21,<br>20	17,<br>21	16,<br>24	14,<br>30	7, 8<br>(3^{d})	6, 6<br>(3^{d})	5, 7<br>(3^{d})	2, 1<br>(4th)	0, 0<br>(-)	5, 4<br>(4th)	7, 8<br>(4th)	8, 1<br>(4th)	4, 6<br>(4th)
yfp Δ20[c, d]										5, 8<br>(1st)	7, 6<br>(1st)	1, 2<br>(1st)	4, 3<br>(4th)	5, 3<br>(4th)	4, 2<br>(4th)	7, 8<br>(3^{d})	2, 1<br>(3^{d})	1, 1<br>(3^{d})
yfp Δ2240[e]	12	9	10	17	9	11	10	16	13	3<br>(3^{d})	4<br>(3^{d})	3<br>(3^{d})	0<br>(-)	0<br>(-)	0<br>(-)	7<br>(4th)	5<br>(4th)	44<br>(4th)
rbcL-FLAG[f]	7	18	9	7	9	7	8	9	11	9<br>(4th)	7<br>(4th)	11<br>(4th)	5<br>(4th)	7<br>(4th)	6<br>(4th)	13<br>(4th)	11<br>(4th)	18<br>(4th)
NS1 Δ30	11	10	-	9	13	-	7	21	-	21<br>(3^{d})	34<br>(2nd)	-	5<br>(3^{d})	11<br>(3^{d})	-	8<br>(3^{d})	14<br>(6th)	-
NS3 Δ30	7	15	-	10	13	-	3	11	-	11<br>(2nd)	2<br>(2nd)	-	2<br>(3^{d})	1<br>(3^{d})	-	11<br>(2nd)	8<br>(2nd)	-
NS4 Δ30	5	14	-	12	14	-	9	7	-	23<br>(2nd)	22<br>(2nd)	-	5<br>(3^{d})	9<br>(3^{d})	-	33<br>(3^{d})	6<br>(3^{d})	-

[a] All colonies from plates without theophylline were calculated from the 6th spot in the dilution series (representing a 5^5 dilution).
[b] The spot on 0.25 mM theophylline spot plates which the number of colonies were counted for is indicated in brackets. (The dilution in these spots is 5^{n-1}, n being the spot number).
[c] The two stacked values per cell is the data for technical duplicates.
[d] This row denotes data for inductions performed using 0.5 mM theophylline.
[e] The 0.25 mM theophylline data was not calculated from spot plates due to a lack of colonies. It was instead calculated from the full-sized induction plates. 40 µl was plated on full-sized plates, i.e. 10x more than the 4 µl for the spots on the spot plates; this was accounted for in the performed calculations. The dilution that was plated and counted is still indicated in brackets.

Sequences

P_{conII}: (TSS in bold)
ACCGGTTTCGAATTGACAATTAATCATCGGCTCGTATAATGGTAC**C**

P_{trc}: (TSS in bold, constant region as in Nakahira et al.[7] is underlined)**:**
AAATATTCTGAAATGAGCTGTTGACAATTAATCATCCGGCTCGTATAATGTGT
GG<u>**A**ATTGTGAGCGGATAACAATTTCATACGCTCACAATTGGTACC</u>

Riboswitch B: (as in Topp et al.[8], aptamer in bold, start codon underlined)
GGTGATACCAGCATCGTCTTGATGCCCTTGGCAGCACCCGCTGCGCAGGG
GGTATCAACAAG<u>ATG</u>

Riboswitch C: (as in Topp et al.[8], aptamer in bold, start codon underlined)
TGATAAGATAGG**GGTGATACCAGCATCGTCTTGATGCCCTTGGCAGCACC**
AAGGGACAACAAG<u>ATG</u>

Riboswitch E*: (as in Topp et al.[8], aptamer in bold, start codon underlined)
GGTGATACCAGCATCGTCTTGATGCCCTTGGCAGCACCCTGCTAAGGAGG
CAACAAG<u>ATG</u>

P_{nrsB}: (native to S6803, truncated at 5'-end to remove -10-box and TSS mapped for *nrsR* expression going in the opposite direction, the two mapped TSS for *nrsBACD* expression are shown in bold).[9]
GTCTGATCTTAGCGGGGGAAGGAGATTTTCACCTGAATTTCATACCCCCT**T**TG
GCAGACTGGGAAA**A**TCTTGGACAAATTCCCAATTTGAGGTGGT

P_{nrsD}: (native to S6803, two mapped TSS in bold).[10]
TATTCGATTCAGTACCAAGTACTATTGCGGGGACAGGACGTTTCTCAAGGCCC
TCATCAATATCCCCCCTGGGGGCATAGAATAGAGAT**C**AAT**T**TTCTACCCCAA
ACCCCCACA

Supporting methods - Using the inducible CRISPR/Cas9-system

For an overview of the CRISPR/Cas9 target vector construction and subsequent workflow in S6803, see Figure 1 in the main text.

Theophylline inducer stock: preparation and use

A 200 mM theophylline stock, dissolved in 100% DMSO, is used. This does not dissolve at RT and requires heating before use. Theophylline is very stable[11] and repeated heating was not found to reduce the stocks performance. To dissolve it, heat in a water bath at 42-50°C, mix a few times by vortexing. When fully dissolved, use directly to make inducer-supplemented BG11-plates (a final concentration of 0.25 mM is recommended. Store at 4°C between uses.

Available pPMQAK1-CRISPR/Cas9 base vectors

The best performing pPMQAK1-CRISPR/Cas9 base vectors described in this study will be submitted to Addgene. These are the constructs in order of expression strength: [E*](104) > [B] > [B]-LVA, all three are supplemented with the Ni^{2+}-inducible P_{nrsB}-*mazF*-LVA curing system.

Designing and constructing sgRNAs

Identification of suitable protospacers in the target area can be done with e.g. Benchling.[3] Useful guidelines are to select spacers that target, if possible, the template strand,[12] that guide Cas9 to cut as close to the edit site as possible,[13] that don't have significant off-target binding, and that don't have extreme GC-content (>75%, <25%). In this study, spacers were also chosen to have an A at the TSS (often by adjusting spacer length by a few nts), however it is unknown if this is strictly necessary for P_{BBa_J23117}.

An sgRNA sequence example:
TTGACAGCTAGCTCAGTCCTAGGGATTGTGCTAGCT**A**TGGCCGATAAGCAGA
AGAATGTTTTAGAGCTAGAAATAGCAAGTTAAAATAAGGCTAGTCCGTTATC
AACTTGAAAAAGTGGCACCGAGTCGGTGCTTTTTTT
(Blue: P_{BBa_J23117}, bold: TSS, underlined: spacer, green: Cas9-handle, yellow: *S. pyogenes* terminator).

To make the sgRNA piece compatible for BsaI-based Golden Gate cloning, the following overhangs are required on the 5'- and 3'-ends of the above sgRNA. BsaI-site shown in bold, created overhangs upon digestion are underlined.

- Fwd primer overhang: 5'-ACACGT**GGTCTC**GGTTT-….
- Rev primer overhang: 5'-AACGTC**GGTCTC**GCGAA-….

For a multiple-target construct

When multiplexing, an sgRNA-array needs to be constructed and this sgRNA-array must have the same BsaI-containing overhangs as described above. In this study this was done using the method described by Li et al.[2] The following template vectors built in this study were used for this purpose: pMD19-BsaI- P_{BBa_J23117}-sgRNA-BsaI-Sp^r and pMD19-Cas9_handle-*S.pyogenes*_terminator- P_{BBa_J23117}-Cm^r.

Designing and constructing Donor DNAs

In this study, homology arms of roughly 350 bp were used on either side of the intended edit. However, for e.g. large insertions it could be beneficial to use longer homology arms.[2]

Donor DNAs are preferably constructed by overlap-PCR to join the two homology arms together to one piece. To create the desired edit, the primers used to amplify the homology regions are designed accordingly. The donor DNA must also be designed to mutate or remove the PAM and preferably also parts of the proximal seed-sequence of the protospacer. See the study by Jiang et al. for advice on which mutations are effective.[14] For larger insertions the donor DNA segments can be added as separate pieces to the Golden Gate assembly.

To make the donor DNA piece compatible for BsaI-based Golden Gate cloning, the following overhangs are required on the outermost 5'- and 3'-ends. BsaI-site shown in bold, created overhangs upon digestion are underlined.

- Fwd primer overhang: 5'-AACGTC**GGTCTC**GTTCG-….
- Rev primer overhang: 5'-ACACGT**GGTCTC**GGCCA-….

For a multiple-target construct

For multiplexed targeting the above overhangs must be used for the forward primer of the first donor DNA, and the reverse primer for the last donor DNA, respectively. The rest of the primers must be designed (e.g. with Benchling)[3] to have BsaI-overhangs that allow for assembly of the different donor-DNAs in the desired order.

Golden Gate assembly of the pPMQAK1-CRISPR/Cas9 target vector
All parts (sgRNA, donor DNA, and pPMQAK1-CRISPR/Cas9 base vector) are mixed with BsaI and T4 DNA ligase in a one-pot reaction. A 10 µl reaction is often enough; the volume can be increased to accommodate for e.g. assembly of many fragments for a multiplexing target vector.

- 20 ng pPMQAK1-CRISPR/Cas9 base vector. (No prior digestion is needed!)
- Add a 2:1 molar ratio of each insert (sgRNA, donor DNA).
- Add water so that the total final reaction volume reaches 10 µl (or as decided).
- Add the T4 DNA ligase buffer.
- Add the BsaI and T4 DNA ligase, usually 0.5-1 µl each per reaction.
- Mix and place in the thermocycler (see below).

In the thermocycler, 30 cycles are noted below. However, fewer cycles will also work but yield more background colonies.

1. 37°C – 5 min (digestion)
2. 16°C – 10 min (ligation)
3. Cycle back to step 1+2, x30
4. 55°C – 5 min (enzyme inactivation)
5. 10°C ∞

Transform your *E. coli* using 2-5 µl of the Golden Gate reaction. The pPMQAK1-CRISPR/Cas9 vectors are blue/white-screening compatible. For a sufficiently concentrated plasmid preparation (70-100 ng/µl), miniprep 10 ml *E. coli* culture. Confirm that the construct is correct by sequencing (covering the sgRNA and donor DNA).

Transforming S6803 with the pPMQAK1-CRISPR/Cas9 target vector
- Use 5-10 ml (OD_{730} 0.6-1) per electroporation.
- Pellet cells (10 min, 4500 rpm, 4°C), wash in cold sterile water. Repeat a total of three times. To reduce time, wash all cells together.
- Re-suspend washed cells in enough cold sterile water so that 50 µl can be aliquoted per electroporation into separate eppendorf tubes.
- Add up to 5 µl (100-350 ng DNA) of the vector. Incubate on ice for ~10 min before transferring to cooled electroporation cuvettes (2 mm gap).
- Electroporate using a Bio-Rad MicroPulser, setting EC2 (V=2.5 kV), or similar.
- Place cuvettes back on ice.
- Collect cell suspensions from the cuvettes and suspend in 3 ml BG11, recover for 16-24 h under standard growth conditions.
- Prepare selective (+Km) BG11 plates without and with 0.25 mM theophylline. Collect recovered cells, pellet (10 min, 4500 rpm, 4°C) and re-suspend in ~500 µl BG11.
- Plate ~250 µl each on selective plates without and with inducer.

- Incubate under normal growth conditions. Colonies will start appearing after 1 week; allow them to grow a bit bigger for a total of about 10-14 days.

With a functional sgRNA and non-leaky CRISPR/Cas9, no or a few colonies will appear on the inducer-plate, while the no-inducer plate will have many colonies. If any colonies appear on the inducer-plate, screen these for the desired edit.

Inducing CRISPR/Cas9-mediated genome editing
From the non-inducer transformation plates, pick healthy colonies but avoid the largest colonies due to the risk of these having mutated the CRISPR/Cas9 system. Try to avoid doing pre-cultures as this can enrich cells that have "escaped" the CRISPR/Cas9-system.

- Prepare selective BG11 plates with 0.25 mM theophylline.
- The colonies, preferably triplicates, are picked and suspended in ~50 µl each.
- Prepare dilutions, e.g. a three-step 5x dilution-series, which gives 5x, 25x, and 125x dilution suspensions. A final volume of 40-60 µl of each is enough.
- Plate the suspensions/dilutions (see strategy below), ~40-60 µl (i.e. the whole available volume) per section.
- To control that the induced colonies harbor an active CRISPR/Cas9-system, one can do a spot assay on plates with and without inducer. Equal survival on both plate-types indicates a likely "escaped" mutated colony.
- Incubate for around 10-14 days, until large green colonies are available.

Since the editing efficiency is unknown for new targets, 2-3 different dilutions of the suspended cells can be plated on inducer-plates (see picture below). If a difficult edit is anticipated (e.g. multiplexed editing or large insertion, see right plate below), plate the remaining no-dilution suspension and 5x dilution. If an easier edit is expected (e.g. a small deletion, see left plate below) one can instead plate the 5x-125x suspensions.

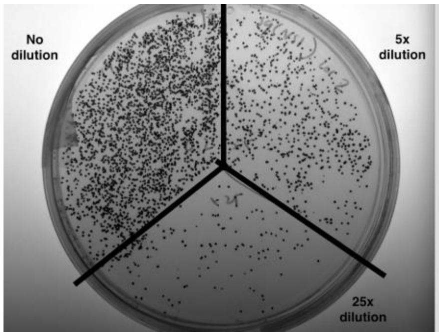

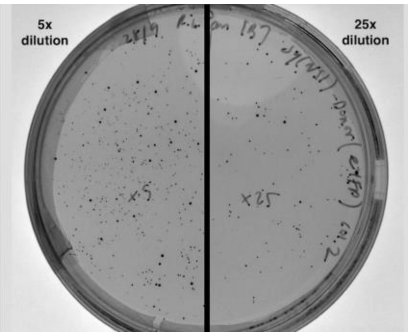

Screening for genome editing
For colony-PCR, ensure that at least one primer binds outside the homology regions used in the donor DNA to avoid false positives. Pick and screen colonies with a healthy phenotype. If multiple transformants were induced, screen colonies from inducer-plates where cells appear to not harbor an inactive CRISPR/Cas9-system (e.g. due to a cell-lawn, or consult a prepared spot assay). Any colonies with un-segregated edits can be streaked on new inducer-plates so that new single colonies will form.

Colonies picked for screening can preferably also be streaked on new non-inducer plates, so that any positives are readily available for the subsequent curing step.

Curing pPMQAK1-CRISPR/Cas9 from edited cells

The curing is performed on non-selective (no Km) BG11 plates supplemented with 10 µM Ni^{2+}.

- Suspend cells from edited colonies in a small volume of BG11 (~80 µl).
- Spread all this on one plate, or if triplicate colonies are cured on one plate, spread ~30 µl per section.
- Incubate plates until colonies appear (~7-10 days).
- Screen colonies for plasmid loss, colony-PCR is preferred as it picks up on small traces of vector better than just streaking on Km-plates.

References

(1) Vasudevan, R., Gale, G. A. R., Schiavon, A. A., Puzorjov, A., Malin, J., Gillespie, M. D., Vavitsas, K., Zulkower, V., Wang, B., Howe, C. J., Lea-Smith, D. J., and McCormick, A. J. (2019) CyanoGate: A Modular Cloning Suite for Engineering Cyanobacteria Based on the Plant MoClo Syntax. *Plant Physiol. 180*, 39–55.

(2) Li, Y., Lin, Z., Huang, C., Zhang, Y., Wang, Z., Tang, Y., Chen, T., and Zhao, X. (2015) Metabolic engineering of Escherichia coli using CRISPR-Cas9 meditated genome editing. *Metab. Eng. 31*, 13–21.

(3) (2021) Benchling [Biology Software].

(4) Doench, J. G., Fusi, N., Sullender, M., Hegde, M., Vaimberg, E. W., Donovan, K. F., Smith, I., Tothova, Z., Wilen, C., Orchard, R., Virgin, H. W., Listgarten, J., and Root, D. E. (2016) Optimized sgRNA design to maximize activity and minimize off-target effects of CRISPR-Cas9. *Nat. Biotechnol. 34*, 184–191.

(5) Calvo-Villamañán, A., Ng, J. W., Planel, R., Ménager, H., Chen, A., Cui, L., and Bikard, D. (2020) On-target activity predictions enable improved CRISPR-dCas9 screens in bacteria. *Nucleic Acids Res. 48*, e64.

(6) Xiao, A., Cheng, Z., Kong, L., Zhu, Z., Lin, S., Gao, G., and Zhang, B. (2014) CasOT: a genome-wide Cas9/gRNA off-target searching tool. *Bioinformatics 30*, 1180–1182.

(7) Nakahira, Y., Ogawa, A., Asano, H., Oyama, T., and Tozawa, Y. (2013) Theophylline-dependent riboswitch as a novel genetic tool for strict regulation of protein expression in cyanobacterium synechococcus elongatus PCC 7942. *Plant Cell Physiol. 54*, 1724–1735.

(8) Topp, S., Reynoso, C. M. K., Seeliger, J. C., Goldlust, I. S., Desai, S. K., Murat, D., Shen, A., Puri, A. W., Komeili, A., Bertozzi, C. R., Scott, J. R., and Gallivan, J. P. (2010) Synthetic riboswitches that induce gene expression in diverse bacterial species. *Appl. Environ. Microbiol. 76*, 7881–7884.

(9) López-Maury, L., García-Domínguez, M., Florencio, F. J., and Reyes, J. C. (2002) A two-component signal transduction system involved in nickel sensing in the cyanobacterium Synechocystis sp. PCC 6803. *Mol. Microbiol. 43*, 247–56.

(10) Foster, A. W., Patterson, C. J., Pernil, R., Hess, C. R., and Robinson, N. J. (2012) Cytosolic Ni(II) sensor in cyanobacterium: nickel detection follows nickel affinity across

four families of metal sensors. *J. Biol. Chem. 287*, 12142–51.
(11) Deshmukh, VN and Jadhav, JK and Sakarkar, D. (2014) Formulation and in vitro evaluation of theophylline anhydrous bioadhesive tablets. *Asian J. Pharm. Free full text Artic. from Asian J Pharm 3*.
(12) Clarke, R., Heler, R., MacDougall, M. S., Yeo, N. C., Chavez, A., Regan, M., Hanakahi, L., Church, G. M., Marraffini, L. A., and Merrill, B. J. (2018) Enhanced Bacterial Immunity and Mammalian Genome Editing via RNA-Polymerase-Mediated Dislodging of Cas9 from Double-Strand DNA Breaks. *Mol. Cell 71*, 42-55.e8.
(13) Paquet, D., Kwart, D., Chen, A., Sproul, A., Jacob, S., Teo, S., Olsen, K. M., Gregg, A., Noggle, S., and Tessier-Lavigne, M. (2016) Efficient introduction of specific homozygous and heterozygous mutations using CRISPR/Cas9. *Nature 533*, 125–129.
(14) Jiang, W., Bikard, D., Cox, D., Zhang, F., and Marraffini, L. A. (2013) RNA-guided editing of bacterial genomes using CRISPR-Cas systems. *Nat Biotechnol 31*, 233–239.
